# cFLIP suppresses caspase-1- and MLKL-independent perinatal lethality driven by auto-processing impaired caspase-8 D387A

**DOI:** 10.1038/s41418-025-01650-0

**Published:** 2025-12-12

**Authors:** Kim Newton, Katherine E. Wickliffe, Allie Maltzman, Debra L. Dugger, Juan Reyes Jr, Natasha Bacarro, Søren Warming, Neha Rohatgi, Rohit Reja, Joshua D. Webster, Vishva M. Dixit

**Affiliations:** 1 Department of Physiological Chemistry, Genentech, South San Francisco, CA USA; 2 Department of Animal Genetic Technologies, Genentech, South San Francisco, CA USA; 3 Roche Informatics, Hoffman-La Roche Canada, Mississauga, ON Canada; 4 Genentech Computational Sciences, Genentech, South San Francisco, CA USA; 5 Department of Pathology, Genentech, South San Francisco, CA USA

**Keywords:** Proteolysis, Development

## Abstract

Death ligands, including FAS ligand (FASL) and tumor necrosis factor (TNF), trigger apoptosis by promoting caspase-8 dimerization and activation. Impaired FAS signaling causes unconventional lymphocytes to accumulate, resulting in lymphadenopathy. Although autoprocessing of caspase-8 is considered important for apoptosis, autoprocessing-deficient *Casp8*^*D387A/D387A*^ mice do not develop lymphadenopathy. We show that this is because heterodimers of caspase-8 D387A and cFLIP, besides suppressing MLKL-driven necroptosis, can also induce apoptosis. Interestingly, caspase-8 D387A elicited MLKL- and caspase-1-independent intestinal atrophy and perinatal lethality in mice lacking cFLIP. Caspase-8 D387A interacted with FADD and RIPK1 in the intestine, where there was aberrant cleavage of N4BP1 and caspase-3, plus enhanced NF-κB signaling. Eliminating FADD, the adaptor protein that promotes caspase-8 oligomerization, prevented this perinatal lethality. Collectively, our results suggest that cFLIP forms heterodimers with caspase-8 D387A to promote apoptosis in some contexts, while limiting the activity of caspase-8 D387A homodimers in others.

## Introduction

The aspartate-specific cysteine protease caspase-8 is an important regulator of cell death and inflammation. It is recruited to intracellular signaling complexes after activation of death receptors (DRs) or certain pattern recognition receptors [1]. In most, but not all instances [2, 3], one of two death effector domains (DEDs) in the N-terminal pro-domain of the caspase-8 zymogen interacts with the adaptor protein FADD [4–6]. This interaction nucleates helical DED filaments that incorporate caspase-8 and its pseudoprotease paralog cFLIP [7, 8]. Homodimerization of caspase-8 and autoprocessing between the two C-terminal catalytic subunits at Asp387 in mouse caspase-8 (Asp374 or Asp384 in human caspase-8) yields the fully active enzyme, which then cleaves at Asp residues between the pro-domain and the larger of the two catalytic subunits [9–11]. The resulting heterotetramer has two active sites, each one formed by a large and a small catalytic subunit. Heterodimers of caspase-8 and the long isoform of cFLIP (cFLIP_L_) also exhibit proteolytic activity but the active site is less dependent on autoprocessing between the caspase-8 catalytic subunits [12, 13]. Shorter isoforms, cFLIP_S_ and cFLIP_R_, largely consist of the DEDs [14, 15] and inhibit caspase-8 activation by perturbing the structure of the helical DED filaments [8].

Caspase-8 cleavage of RIPK1, a kinase often found in caspase-8 signaling complexes, suppresses cell death [16–18]. RIPK1 cleavage may destabilize the signaling complex and thereby limit the extent of caspase-8 activation. Accordingly, mutation of the cleavage site in RIPK1 enhances caspase-8 cleavage of cFLIP_L_ [16]. If caspase-8 activation is transient because RIPK1 is cleaved, then presumably caspase-8 cleaves too little caspase-3, caspase-7, and BID for apoptosis [19–21]. RIPK1 cleavage also prevents interactions between RIPK1 and RIPK3 that promote RIPK3 activation, phosphorylation of MLKL, and necroptosis. Genetic studies highlight the importance of caspase-8 proteolytic activity in suppressing necroptosis. Mice expressing inactive caspase-8 exhibit RIPK3- and MLKL-dependent embryonic lethality [16, 22] similar to mice lacking FADD or caspase-8 [ref. 23–25].

Caspase-8 cleavage of N4BP1 enhances the expression of many proinflammatory genes [26]. The caspase-8 scaffold also contributes to proinflammatory gene expression by FAS and the TRAIL receptors [27–30]. Loss of these proinflammatory functions of caspase-8 may contribute to immunodeficiency in humans homozygous for mutations encoding FADD C105W, caspase-8 Q220R or caspase-8 R248W [31–33]. The mutant proteins appear less stable than their wild-type (WT) counterparts, with the caspase-8 mutant residues residing in the larger catalytic subunit. Individuals exhibit autoimmune lymphoproliferative syndrome (ALPS), susceptibility to infections, and early onset inflammatory bowel disease [31–33]. ALPS was first described in humans with mutations in *FAS* [34, 35]. Patients exhibit autoimmunity, splenomegaly, and lymphadenopathy, accumulating unconventional TCRαβ^+^ CD3^+^ CD4^-^ CD8^-^ CD45RA^+^ T cells [36]. CD3^+^ B220^+^ T cells also accumulate in lymphoproliferation (*lpr*) mutant mice lacking FAS [37], generalized lymphoproliferative disease (*gld*) mutant mice expressing mutant FAS ligand [38], *Casp8*^*-/-*^ *Ripk3*^*-/-*^ mice [23, 24], *Casp8*^*-/-*^ *Mlkl*^*-/-*^ or *Fadd*^*-/-*^ *Mlkl*^*-/-*^ mice [25], and *Cd4*.Cre *Casp8*^*fl/fl*^ *Ripk3*^*-/-*^ mice [39]. Splenomegaly and lymphadenopathy in ALPS and LPR disease is attributed to impaired apoptosis signaling.

The relative contributions of caspase-8 homodimers and caspase-8/cFLIP_L_ heterodimers to cell viability have been explored using mice expressing caspase-8 D387A [16, 40, 41] or caspase-8 F122G, L123G [41]. Caspase-8 D387A cannot be cleaved between the catalytic subunits and only exhibits detectable activity as a heterodimer with cFLIP_L_. The F122G, L123G mutations within the caspase-8 pro-domain support heterodimer but not homodimer interactions. These “homodimer-deficient” mice are viable, unlike *Casp8*^*-/-*^ mice, consistent with caspase-8/cFLIP_L_ heterodimers suppressing necroptosis [24]. Although their thymocytes exhibit reduced FAS-induced apoptosis, the mice do not get LPR disease [16, 40, 41]. In this study, we characterize *Casp8*^*D387A/D387A*^ *Cflar*^*-/-*^ mice, which should lack both caspase-8 heterodimer and homodimer activity.

## Results

### cFLIP deficiency causes lethality in *Casp8*^*D387A/D387A*^ mice

**Fig. 1:**
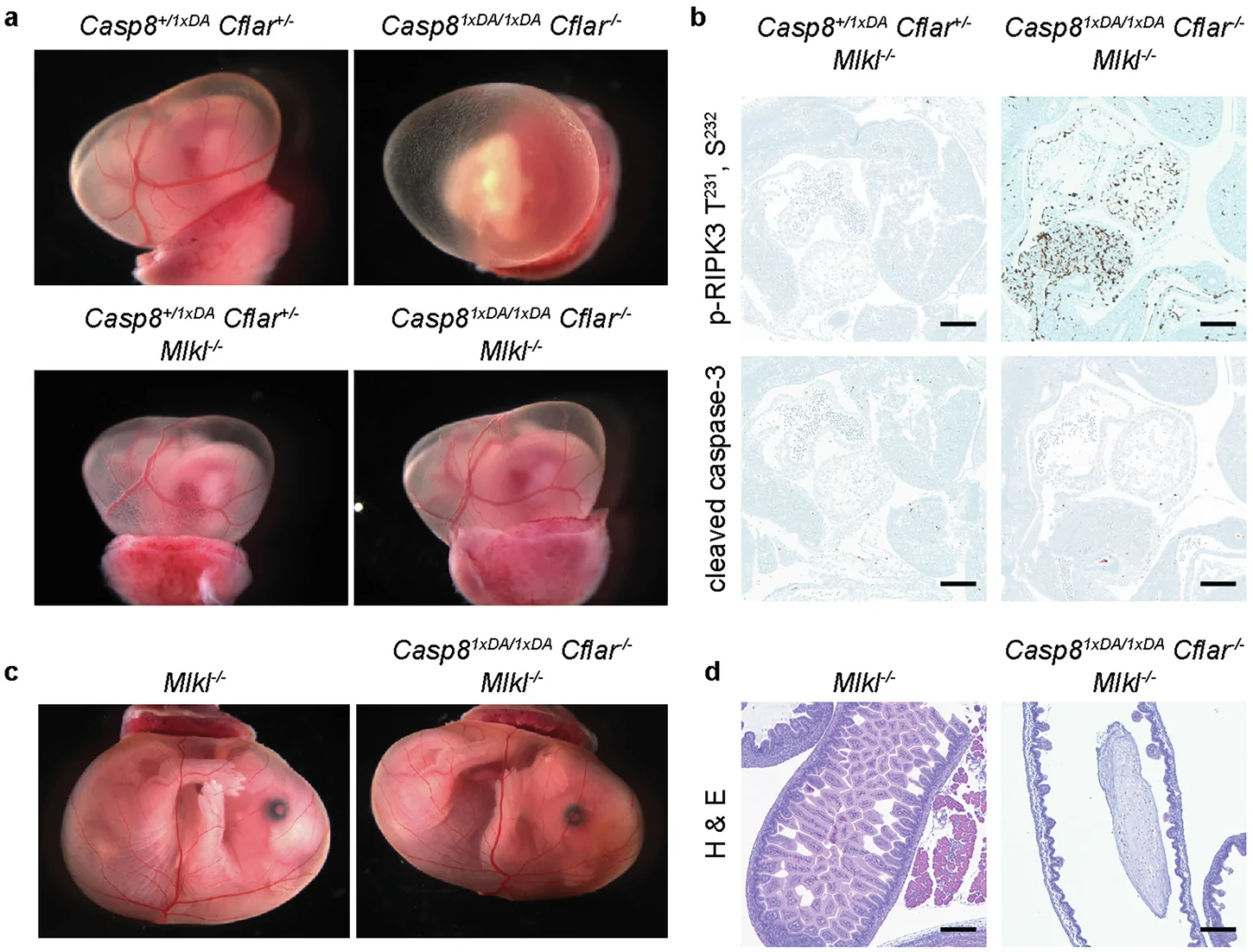
*Casp8*^*1xDA/1xDA*^*Cflar*^*-/-*^ mice die during embryogenesis from aberrant necroptosis. **a** E10.5 embryos. Results representative of 5 *Casp8*^*+/1xDA*^ *Cflar*^*+/-*^, 3 *Casp8*^*1xDA/1xDA*^ *Cflar*^*-/-*^, 4 *Casp8*^*+/1xDA*^ *Cflar*^*+/-*^ *Mlkl*^*-/-*^, and 5 *Casp8*^*1xDA/1xDA*^ *Cflar*^*-/-*^ *Mlkl*^*-/-*^ embryos. **b** E10.5 embryo sections immunolabelled for phospho-RIPK3 T^231^, S^232^ or cleaved caspase-3 (brown). Scale bar, 200 μm. Results representative of 3 *Casp8*^*+/1xDA*^ *Cflar*^*+/-*^ *Mlkl*^*-/-*^ and 5 *Casp8*^*1xDA/1xDA*^ *Cflar*^*-/-*^ *Mlkl*^*-/-*^ embryos. **c** E17.5 embryos. Results representative of 5 *Mlkl*^*-/-*^ and 8 *Casp8*^*1xDA/1xDA*^ *Cflar*^*-/-*^ *Mlkl*^*-/-*^ analyzed at E17.5 or E18.5. **d** E18.5 intestine sections stained with hematoxylin and eosin (H & E). Scale bar, 200 μm. Results representative of 2 *Mlkl*^*-/-*^ and 2 *Casp8*^*1xDA/1xDA*^ *Cflar*^*-/-*^ *Mlkl*^*-/-*^ embryos.

To determine if caspase-8/cFLIP_L_ heterodimers mediate extrinsic apoptosis and thereby suppress LPR disease, we disrupted the *Cflar* gene encoding cFLIP in *Casp8*^*D387A/D387A*^ mice (also called *Casp8*^*1xDA/1xDA*^ mice) [16]. *Cflar*^*-/-*^ mice die around E9.5 [ref. 42]. Lethality is attributed to aberrant activation of FADD- and caspase-8-dependent apoptosis because *Cflar*^*-/-*^ *Fadd*^*-/-*^ *Ripk3*^*-/-*^ mice are viable, but *Cflar*^*-/-*^ *Ripk3*^*-/-*^ mice are not [43]. *Casp8*^*1xDA/1xDA*^ *Cflar*^*-/-*^ mice were dead by E10.5 and exhibited disruption of the yolk sac vasculature (Fig. 1a), similar to *Casp8*^*-/-*^ mice [44] and mice expressing catalytically inactive caspase-8 C362A or C362S [16, 22]. Necroptosis drove this lethality because *Casp8*^*1xDA/1xDA*^ *Cflar*^*-/-*^ *Mlkl*^*-/-*^ embryos were viable at E10.5 (Fig. 1a), but contained phospho-RIPK3 T^231^, S^232^ (Fig. 1b), indicative of necroptosis signaling [45]. Cleaved caspase-3, which induces either apoptosis or pyroptosis [46], was not increased in E10.5 *Casp8*^*1xDA/1xDA*^ *Cflar*^*-/-*^ *Mlkl*^*-/-*^ embryos compared with littermate controls (Fig. 1b). In contrast to *Casp8*^*1xDA/1xDA*^ *Cflar*^*-/-*^ *Mlkl*^*-/-*^ embryos, which appeared grossly normal between E17.5 and E18.5 (Fig. 1c), *Casp8*^*1xDA/1xDA*^ *Cflar*^*-/-*^ *Ripk1*^*D138N/D138N*^ embryos expressing catalytically inactive RIPK1 were dead by E12.5 (Fig. S1a). Therefore, necroptosis in *Casp8*^*1xDA/1xDA*^ *Cflar*^*-/-*^ embryos does not require the kinase activity of RIPK1.

**Fig. 2:**
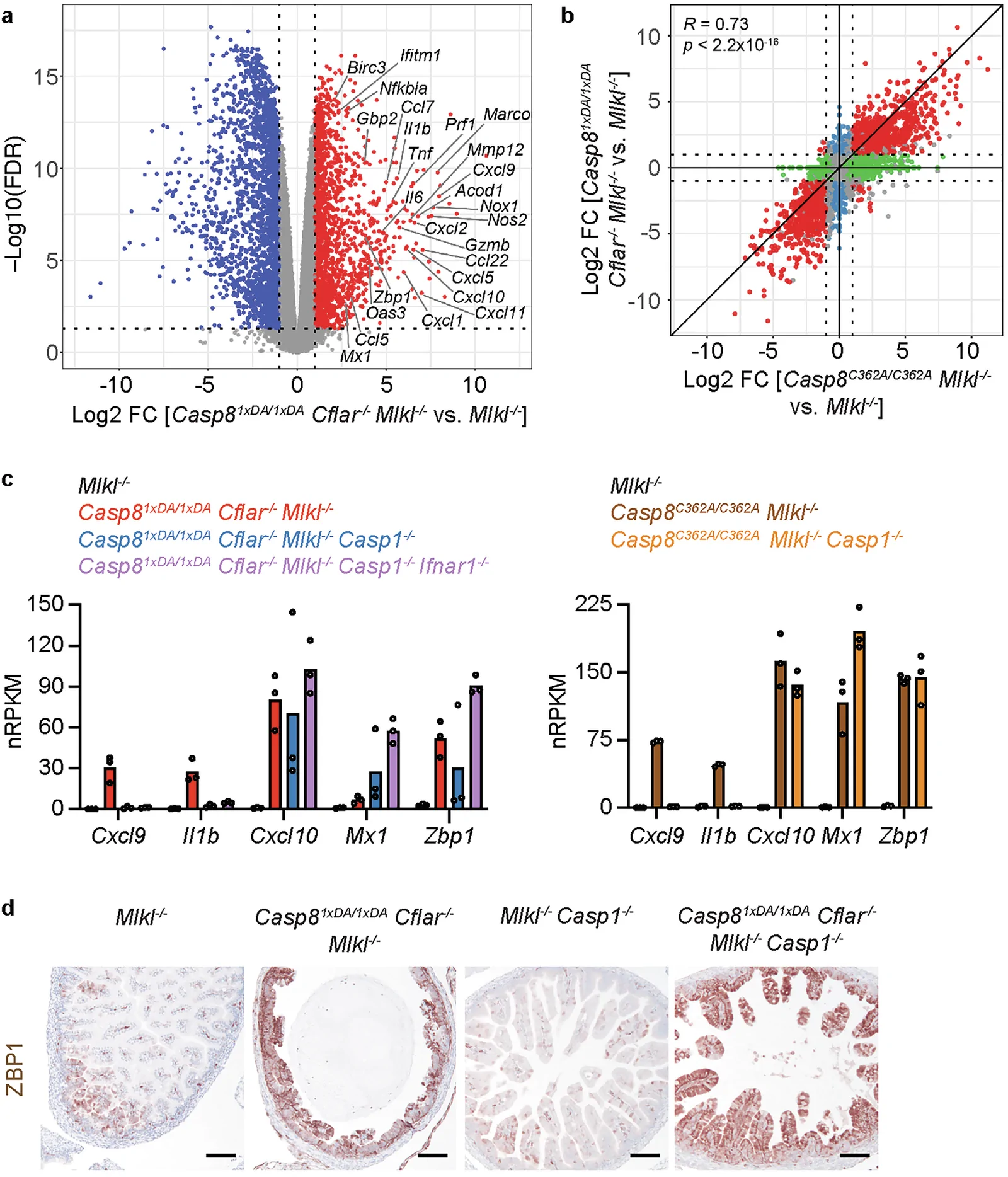
Proinflammatory gene expression in E18.5 *Casp8*^*1xDA/1xDA*^*Cflar*^*-/-*^*Mlkl*^*-/-*^ intestines. **a** Volcano plot indicating differentially expressed genes in E18.5 *Casp8*^*1xDA/1xDA*^ *Cflar*^*-/-*^ *Mlkl*^*-/-*^ intestines (n = 3) compared with *Mlkl*^*-/-*^ littermate intestines (n = 3). FC, fold change. FDR, false discovery rate. Blue dots, downregulated genes. Red dots, upregulated genes. **b** 4-way plot comparing differentially expressed genes in E18.5 *Casp8*^*1xDA/1xDA*^ *Cflar*^*-/-*^ *Mlkl*^*-/-*^ or *Casp8*^*C362A/C362A*^ *Mlkl*^*-/-*^ intestines when compared with *Mlkl*^*-/-*^ littermate intestines (n = 3 per genotype). Red dots, genes dysregulated in both comparisons (FDR < 0.05, Log2 FC > 1 or < −1). Green dots, genes dysregulated only *Casp8*^*C362A/C362A*^ *Mlkl*^*-/-*^ intestines (FDR < 0.05, Log2 FC > 1 or < -1 on x-axis, and FDR ≥ 0.05 on y-axis). Blue dots, genes dysregulated only in *Casp8*^*1xDA/1xDA*^ *Cflar*^*-/-*^ *Mlkl*^*-/-*^ intestines (FDR < 0.05, Log2 FC > 1 or < −1 on y-axis, and FDR ≥ 0.05 on x-axis). **c** Transcript levels in E18.5 intestines. Bars indicate the mean (n = 3 per genotype). Circles, individual embryos. **d** E18.5 intestine sections immunolabelled for ZBP1. Scale bar, 100 μm. Results representative of 2 *Mlkl*^*-/-*^, 2 *Casp8*^*1xDA/1xDA*^ *Cflar*^*-/-*^ *Mlkl*^*-/-*^, 2 *Mlkl*^*-/-*^ *Casp1*^*-/-*^, and 2 *Casp8*^*1xDA/1xDA*^ *Cflar*^*-/-*^ *Mlkl*^*-/-*^ *Casp1*^*-/-*^ embryos.

*Casp8*^*1xDA/1xDA*^ *Cflar*^*-/-*^ *Mlkl*^*-/-*^ mice were not found at 5–7 days after birth (Table 1), indicating perinatal lethality. *Casp8*^*1xDA/1xDA*^ *Mlkl*^*-/-*^ mice are viable [41], indicating that cFLIP loss triggers this lethality. E18.5 *Casp8*^*1xDA/1xDA*^ *Cflar*^*-/-*^ *Mlkl*^*-/-*^ embryos exhibited atrophy of the small intestine (Fig. 1d) and thymus (Fig. S1b, c), and elevated levels of IL-6 and CXCL9 in the intestine (Fig. S1d). RNA sequencing indicated dysregulated expression of 4,323 genes in E18.5 *Casp8*^*1xDA/1xDA*^ *Cflar*^*-/-*^ *Mlkl*^*-/-*^ intestines compared with *Mlkl*^*-/-*^ intestines (Log2 fold change < −1 or >1, FDR [false discovery rate] <0.05). Up-regulated genes included chemokines and cytokines (for example, *Ccl5*, *Ccl7*, *Ccl22*, *Cxcl1*, *Cxcl2*, *Cxcl5*, *Cxcl9*, *Cxcl10*, *Cxcl11*, *Il1b*, *Il6*, and *Tnf*) as well as other genes linked to pathogen defense (for example, *Acod1*, *Gbp2*, *Gzmb*, *Ifitm1, Marco*, *Mmp12*, *Mx1, Nox1*, *Nos2*, *Oas3, Prf1*, and *Zbp1*) (Fig. 2a). The transcriptomes of E18.5 *Casp8*^*1xDA/1xDA*^ intestines and *Mlkl*^*-/-*^ intestines were largely similar (Fig. S1e), suggesting that cFLIP limits aberrant signaling by caspase-8 D387A.

**Table 1:** Actual (expected) offspring numbers from intercrossing *Casp8*^*+/1xDA*^ *Cflar*^*+/-*^ mice.

Genetic Background	Age	*Casp8*^*+/+*^ *Cflar*^*+/+*^	*Casp8*^*+/1xDA*^ *Cflar*^*+/-*^	*Casp8*^*1xDA/1xDA*^ *Cflar*^*-/-*^
*Mlkl*^*-/-*^	E17.5–E18.5	12 (9.25)	15 (18.5)	10 (9.25)
*Mlkl*^*-/-*^	P5–P7	35 (25.5)	67 (51)	0 (25.5)
*Mlkl*^*-/-*^ *Casp1*^*-/-*^	P5–P7	23 (18.25)	50 (36.5)	0 (18.25)
*Mlkl*^*-/-*^ *Casp1*^*-/-*^ *Ifnar1*^*-/-*^	P5–P7	14 (12.25)	35 (24.5)	0 (12.25)
*Mlkl*^*-/-*^ *Fadd*^*-/-*^	P5–P7	7 (8.75)	20 (17.5)	8 (8.75)
*Mlkl*^*-/-*^ *Fadd*^*-/-*^ *Casp1*^*-/-*^	P5–P7	4 (5.5)	11 (11)	7 (5.5)
*Mlkl*^*-/-*^ *Fadd*^*+/-*^ *Casp1*^*-/-*^	P5–P7	15 (15.25)	43 (30.5)	3 (15.25)

*E* embryonic day, *P* postnatal day.

**Fig. 3:**
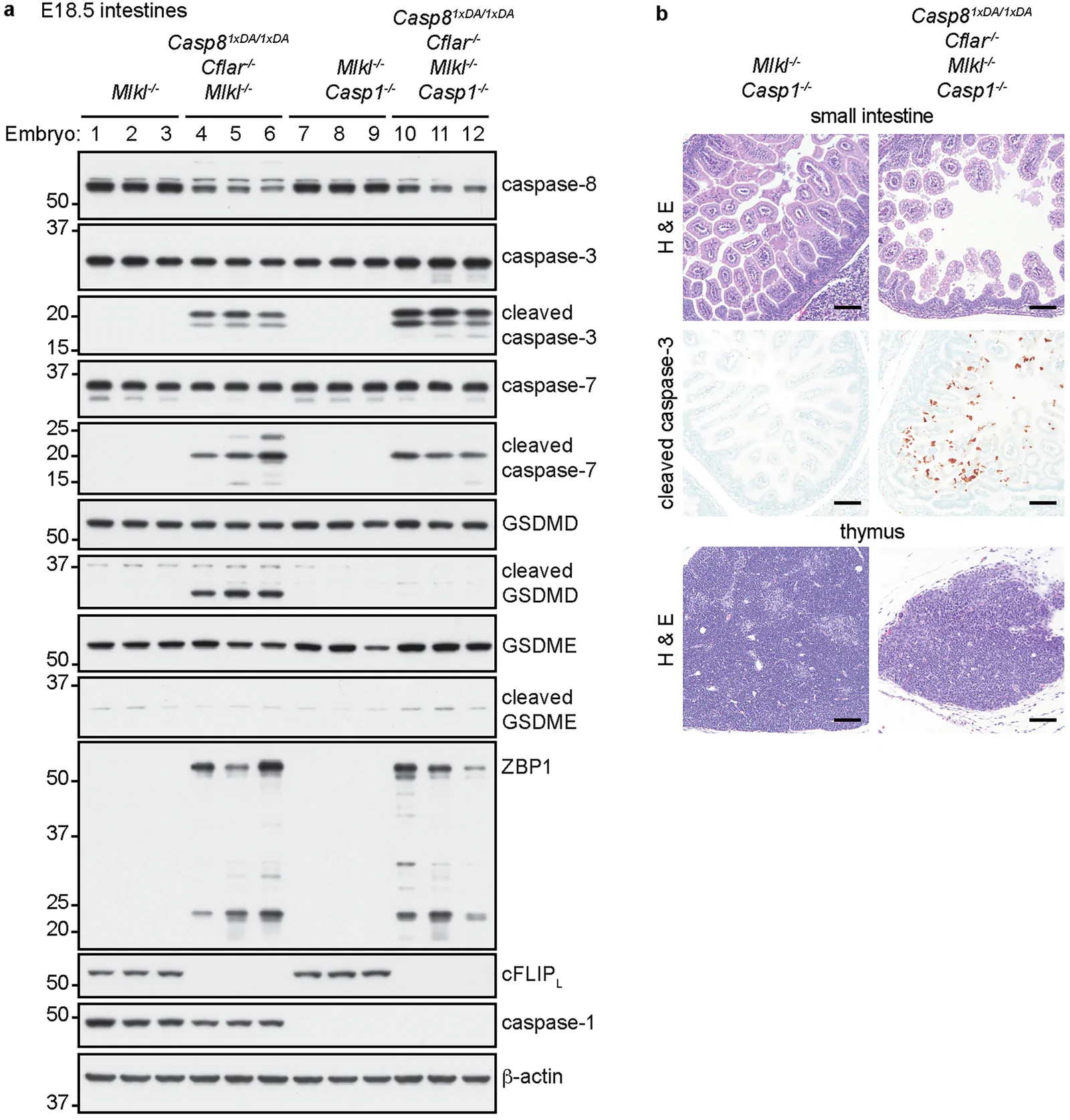
*Casp8*^*1xDA/1xDA*^*Cflar*^*-/-*^*Mlkl*^*-/-*^ mice exhibit caspase-1-independent intestinal atrophy. **a** Western blots of E18.5 intestines. Lanes, individual embryos. **b** E18.5 tissue sections immunolabelled for cleaved caspase-3, or stained with H & E. Scale bar, 200 μm. Results representative of 2 *Mlkl*^*-/-*^ *Casp1*^*-/-*^ and 2 *Casp8*^*1xDA/1xDA*^ *Cflar*^*-/-*^ *Mlkl*^*-/-*^ *Casp1*^*-/-*^ embryos.

*Casp8*^*1xDA/1xDA*^ *Cflar*^*-/-*^ *Mlkl*^*-/-*^ intestinal atrophy resembled that in *Casp8*^*C362A/C362A*^ *Mlkl*^*-/-*^, *Casp8*^*C362S/C362S*^ *Mlkl*^*-/-*^, and *Casp8*^*1xDA/1xDA*^ *Fadd*^*-/-*^ *Mlkl*^*-/-*^ mice, where perinatal lethality is driven, in part, by caspase-1 and its adaptor ASC [22, 41, 47]. Indeed, 1117 and 1356 of the 1901 transcripts up-regulated in E18.5 *Casp8*^*1xDA/1xDA*^ *Cflar*^*-/-*^ *Mlkl*^*-/-*^ intestines were also more abundant in *Casp8*^*C362A/C362A*^ *Mlkl*^*-/-*^ (Fig. 2b) and *Casp8*^*C362A/C362A*^ *Cflar*^*-/-*^ *Mlkl*^*-/-*^ intestines (Fig. S1f), respectively. Some of these transcripts, including *Cxcl9* and *Il1b*, were reduced in E18.5 *Casp8*^*C362A/C362A*^ *Mlkl*^*-/-*^ *Casp1*^*-/-*^ or *Casp8*^*1xDA/1xDA*^ *Cflar*^*-/-*^ *Mlkl*^*-/-*^ *Casp1*^*-/-*^ intestines, suggesting that caspase-1-dependent cell death promoted their expression (Fig. 2c and Fig. S1g). However, other transcripts, including *Cxcl10*, *Mx1*, and *Zbp1*, were not reduced by caspase-1 deficiency, indicating gene expression independent of caspase-1- or MLKL-dependent cell death. We confirmed elevated expression of ZBP1 protein in E18.5 *Casp8*^*1xDA/1xDA*^ *Cflar*^*-/-*^ *Mlkl*^*-/-*^ and *Casp8*^*1xDA/1xDA*^ *Cflar*^*-/-*^ *Mlkl*^*-/-*^ *Casp1*^*-/-*^ intestines by immunolabelling (Fig. 2d) and western blotting (Fig. 3a). *Cxcl10*, *Mx1*, and *Zbp1* are known to be induced by interferon signaling [48–50], but loss of the type I interferon receptor IFNAR1 did not normalize *Cxcl10*, *Mx1* or *Zbp1* expression in E18.5 *Casp8*^*1xDA/1xDA*^ *Cflar*^*-/-*^ *Mlkl*^*-/-*^ *Casp1*^*-/-*^ *Ifnar*^*-/-*^ intestines (Fig. 2c). Therefore, type I interferon signaling is dispensable for proinflammatory gene induction in *Casp8*^*1xDA/1xDA*^ *Cflar*^*-/-*^ *Mlkl*^*-/-*^ intestines.

Although we detected caspase-1-dependent cleavage of GSDMD in E18.5 *Casp8*^*1xDA/1xDA*^ *Cflar*^*-/-*^ *Mlkl*^*-/-*^ intestines (Fig. 3a), eliminating caspase-1 (or caspase-1 plus IFNAR1) did not prevent perinatal lethality (Table 1). Like E18.5 *Casp8*^*1xDA/1xDA*^ *Cflar*^*-/-*^ *Mlkl*^*-/-*^ embryos (Fig. 1d and Fig. S1b–h), E18.5 *Casp8*^*1xDA/1xDA*^ *Cflar*^*-/-*^ *Mlkl*^*-/-*^ *Casp1*^*-/-*^ embryos exhibited aberrant caspase-3/7 cleavage in the intestine (Fig. 3a, b), and atrophy of the small intestine and thymus (Fig. 3b). These data suggest that cFLIP prevents the caspase-8 D387A scaffold from engaging caspase-1-driven pyroptosis, as well as caspase-1-independent signals that elicit processing of caspase-3/7 and intestinal atrophy. Caspase-3 can cleave GSDME to induce pyroptosis, but we did not detect aberrant GSDME cleavage in E18.5 *Casp8*^*1xDA/1xDA*^ *Cflar*^*-/-*^ *Mlkl*^*-/-*^ or *Casp8*^*1xDA/1xDA*^ *Cflar*^*-/-*^ *Mlkl*^*-/-*^ *Casp1*^*-/-*^ intestines (Fig. 3a). We noted that E18.5 *Casp8*^*1xDA/1xDA*^ *Cflar*^*-/-*^ *Mlkl*^*-/-*^ intestines contained less detergent-soluble caspase-8 than WT, *Mlkl*^*-/-*^, *Casp8*^*1xDA/1xDA*^, *Casp8*^*4xDA/4xDA*^, or *Casp8*^*4xDA/4xDA*^ *Mlkl*^*-/-*^ intestines (Fig. 3a and Fig. S2). Mouse embryonic fibroblasts (MEFs) from *Casp8*^*4xDA/4xDA*^ *Mlkl*^*-/-*^ mice, which express caspase-8 D212A, D218A, D225A, D387A with all autoprocessing sites mutated [16], also contained less detergent soluble caspase-8 after *Cflar* deletion (Fig. S5f). Loss of cFLIP may promote detergent-insoluble autoprocessing-impaired caspase-8 complexes.

### Heterodimers of caspase-8 D387A and cFLIP contribute to extrinsic apoptosis

Although caspase-8 D387A homodimers lack detectable proteolytic activity in vitro [13], we wondered if caspase-8 D387A was inducing death receptor-induced apoptosis in E18.5 *Casp8*^*1xDA/1xDA*^ *Cflar*^*-/-*^ *Mlkl*^*-/-*^ intestines. To explore this possibility, we challenged *Casp8*^*1xDA/1xDA*^ *Cflar*^*-/-*^ *Mlkl*^*-/-*^ MEFs with different apoptotic stimuli. As reported [51], defective auto-processing of caspase-8 skewed TNF-induced cell death signaling towards necroptosis. For example, both *Casp8*^*1xDA/1xDA*^ and *Casp8*^*4xDA/4xDA*^ MEFs exhibited less caspase-3 cleavage, but more RIPK3 autophosphorylation than WT MEFs in response to TNF plus translational inhibitor cycloheximide (T + C) (Fig. S3a). *Casp8*^*4xDA/4xDA*^ *Mlkl*^*-/-*^ MEFs were more resistant than *Casp8*^*4xDA/4xDA*^ MEFs to killing by T + C (Fig. S3b), consistent with necroptosis contributing to the death of the latter. Further evidence of impaired autoprocessing of caspase-8 promoting necroptosis was obtained in the TNF model of systemic inflammatory response syndrome [52]. Both *Casp8*^*1xDA/1xDA*^ and *Casp8*^*4xDA/4xDA*^ mice were more susceptible than WT mice to TNF toxicity, whereas *Casp8*^*4xDA/4xDA*^ *Mlkl*^*-/-*^ mice were not (Fig. S3c).

**Fig. 4:**
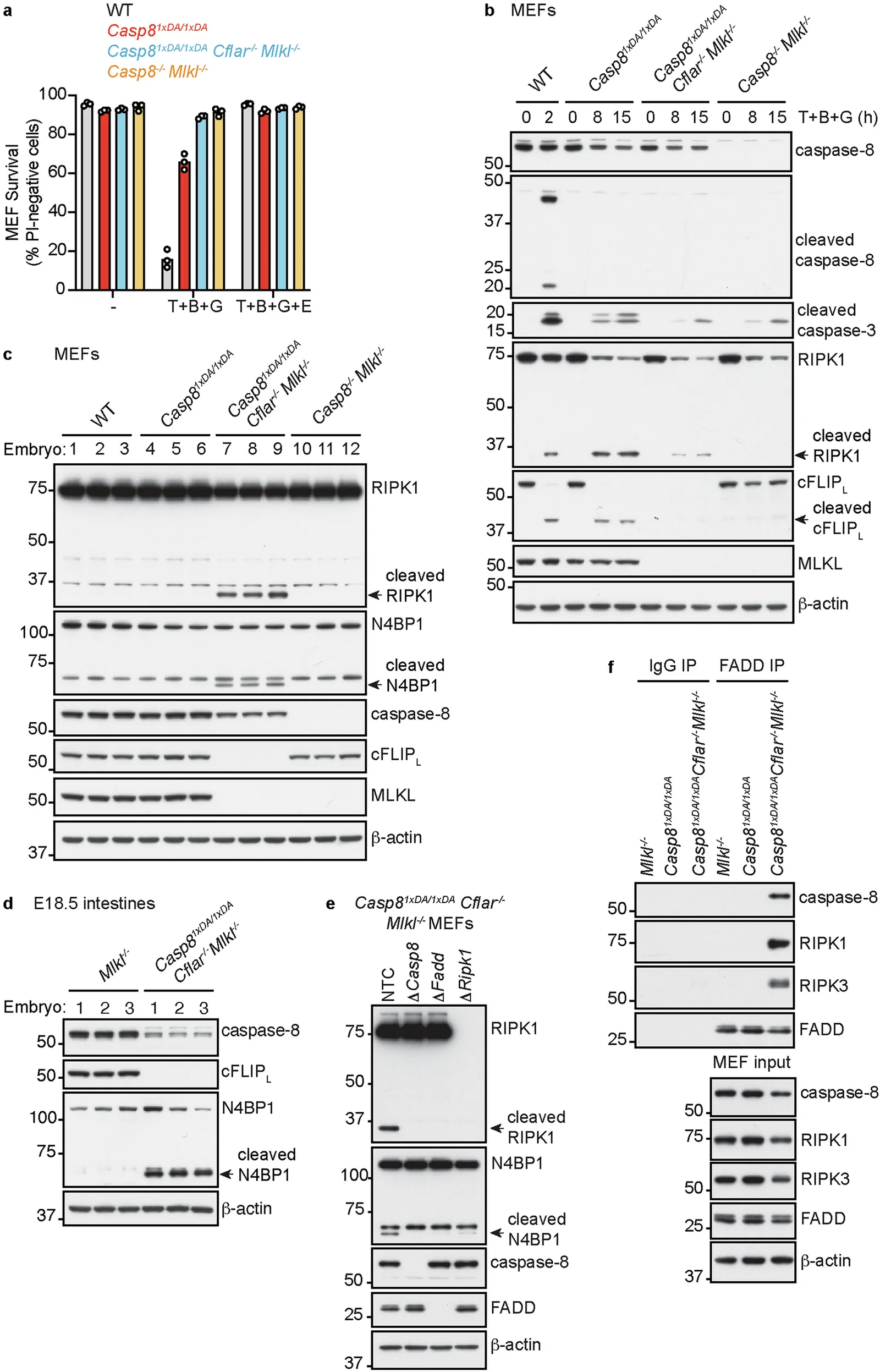
Heterodimers of caspase-8 D387A and cFLIP mediate TNF-induced apoptosis. **a** Percentage of surviving MEFs at 24 h after treatment. Bars indicate the mean. Circles, cells from individual embryos (n = 3 per genotype). T, 100 ng/mL TNF. B, 2 μM BV6. G, 10 μM GSK843. E, 20 μM emricasan. PI, propidium iodide. **b**–**f** Western blots of primary MEFs or their immunoprecipitates (IP). NTC, non-targeting control sgRNA. Lanes (**c**, **d**), cells from individual embryos. Results in (**b**, **e**, **f**) representative of 2 independent experiments.

To limit our analyses to extrinsic apoptosis, we prevented necroptosis using the RIPK3 inhibitor GSK´843 [ref. 53]. TNF, inhibitor of apoptosis protein antagonist BV6, plus GSK´843 (T + B + G) killed *Casp8*^*1xDA/1xDA*^ MEFs, albeit not as robustly as WT MEFs (Fig. 4a). Cell death was deemed apoptotic because annexin V staining preceded secondary plasma membrane rupture and it was prevented by the pan-caspase inhibitor emricasan (Fig. 4a and Fig. S4a, b). Cell death was not due to GSDMD- or GSDME-induced pyroptosis because WT and *Gsdmd*^*-/-*^ *Gsdme*^*-/-*^ MEFs exhibited comparable cell death in response to T + B + G (Fig. S5a, b)*. Casp8*^*1xDA/1xDA*^ *Cflar*^*-/-*^ *Mlkl*^*-/-*^ MEFs were as resistant as *Casp8*^*-/-*^ *Mlkl*^*-/-*^ MEFs to T + B + G (Fig. 4a), but were normally susceptible to the kinase inhibitor staurosporine (Fig. S5c), an inducer of BAX- and BAK-dependent intrinsic apoptosis [54]. These results suggest that caspase-8 D387A/cFLIP heterodimers promote extrinsic apoptosis, whereas caspase-8 D387A homodimers do not. In support of this model, T + B + G induced less cleavage of caspase-3 and RIPK1 in *Casp8*^*1xDA/1xDA*^ *Cflar*^*-/-*^ *Mlkl*^*-/-*^ MEFs compared with *Casp8*^*1xDA/1xDA*^ MEFs (Fig. 4b). However, longer western blot exposures revealed low level, basal processing of both RIPK1 and N4BP1 in *Casp8*^*1xDA/1xDA*^ *Cflar*^*-/-*^ *Mlkl*^*-/-*^ MEFs, which was not evident in wild-type (WT), *Casp8*^*1xDA/1xDA*^, or *Casp8*^*C362A/C362A*^ *Cflar*^*-/-*^ *Mlkl*^*-/-*^ MEFs (Fig. 4c and Fig. S5d). *Cflar* deletion by CRISPR/Cas9 gene editing in *Casp8*^*1xDA/1xDA*^ or *Casp8*^*4xDA/4xDA*^ *Mlkl*^*-/-*^ MEFs was sufficient for RIPK1 and N4BP1 cleavage (Fig. S5e, f), indicating that it is cFLIP loss, not the combined loss of cFLIP and MLKL, that promotes these cleavage events. Processing of N4BP1 was also detected in E18.5 *Casp8*^*1xDA/1xDA*^ *Cflar*^*-/-*^ *Mlkl*^*-/-*^ intestines, but not *Mlkl*^*-/-*^ intestines (Fig. 4d). These data suggest that caspase-8 D387A homodimers retain some degree of proteolytic activity and are held in check by cFLIP.

### cFLIP suppresses NF-κB activation and gene transcription triggered by FADD, RIPK1, and caspase-8 D387A

*Fadd* or *Casp8* deletion prevented RIPK1 and N4BP1 cleavage in *Casp8*^*1xDA/1xDA*^ *Cflar*^*-/-*^ *Mlkl*^*-/-*^ MEFs (Fig. 4e), consistent with FADD orchestrating the assembly of active caspase-8 D387A homodimers. Interestingly, *Ripk1* deletion also suppressed N4BP1 cleavage in *Casp8*^*1xDA/1xDA*^ *Cflar*^*-/-*^ *Mlkl*^*-/-*^ MEFs (Fig. 4e). Inhibiting RIPK1 with Nec1s eliminated basal RIPK1 autophosphorylation in *Casp8*^*1xDA/1xDA*^ *Cflar*^*-/-*^ *Mlkl*^*-/-*^ MEFs but only slightly reduced N4BP1 cleavage (Fig. S5g). These data suggest the RIPK1 scaffold contributes to caspase-8 D387A activation and/or N4BP1 recruitment, while RIPK1 catalytic activity is less critical. Consistent with cFLIP loss promoting the assembly of a caspase-8 D387A signaling complex, caspase-8 and RIPK1 coimmunoprecipitated with FADD from *Casp8*^*1xDA/1xDA*^ *Cflar*^*-/-*^ *Mlkl*^*-/-*^ MEFs, but not *Casp8*^*1xDA/1xDA*^ or *Mlkl*^*-/-*^ MEFs (Fig. 4f).

**Fig. 5:**
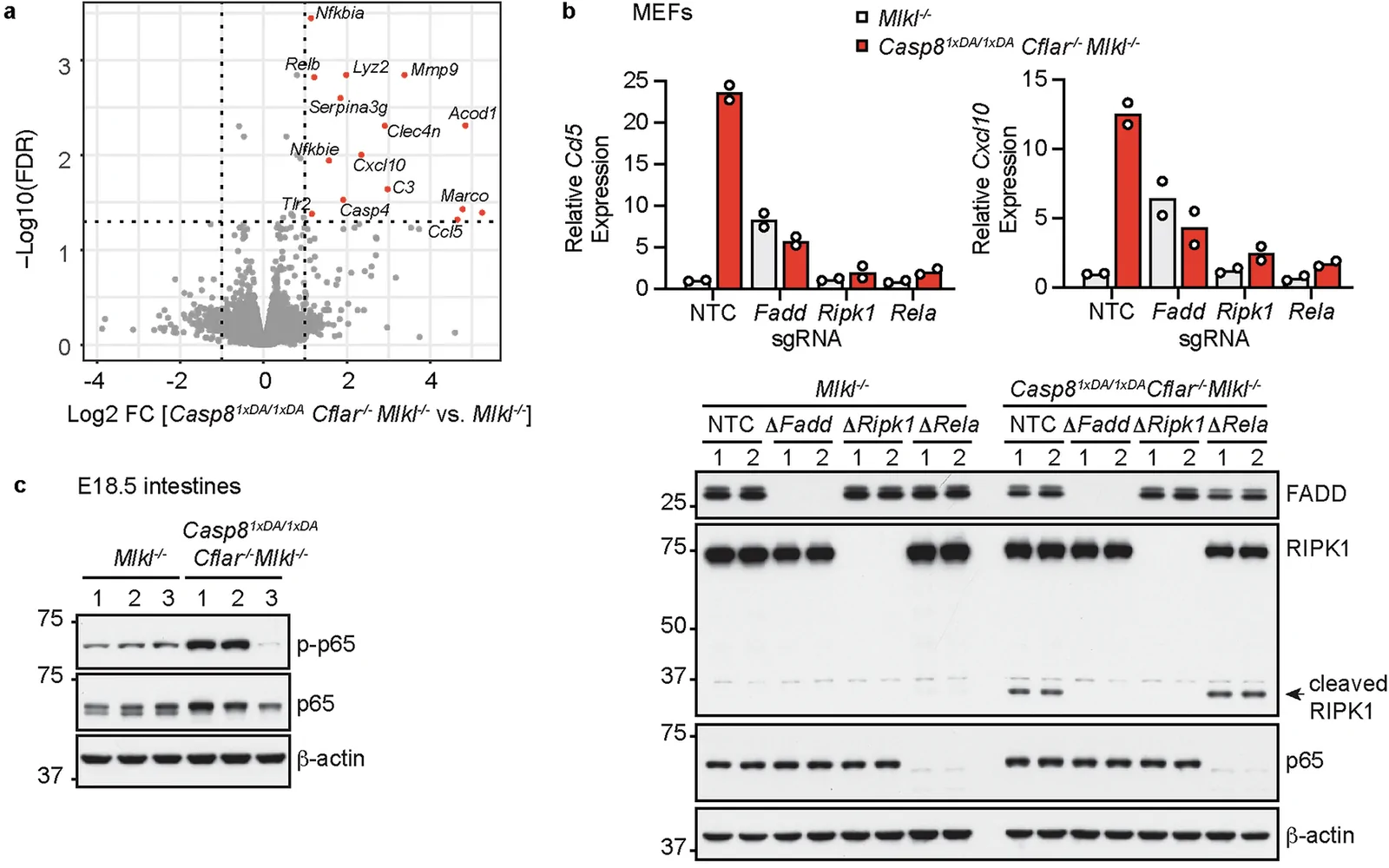
FADD-dependent NF-κB activation and chemokine expression in *Casp8*^*1xDA/1xDA*^*Cflar*^*-/-*^ MEFs. **a** Volcano plot indicates differentially expressed genes in *Casp8*^*1xDA/1xDA*^ *Cflar*^*-/-*^ *Mlkl*^*-/-*^ MEFs compared with *Mlkl*^*-/-*^ MEFs (n = 3 per genotype). Red dots, upregulated genes. FC, fold change. FDR, false discovery rate. **b** Graphs (top) show relative *Ccl5* and *Cxcl10* transcript levels in MEFs characterized by western blot (bottom). Bars indicate the mean. Circles, MEFs from individual embryos. **c** Western blots of E18.5 intestines. Lanes, individual embryos.

Besides increased N4BP1 cleavage, *Casp8*^*1xDA/1xDA*^ *Cflar*^*-/-*^ *Mlkl*^*-/-*^ MEFs exhibited elevated expression of 15 genes when compared with *Mlkl*^*-/-*^ MEFs (Log2 fold change > 1, FDR < 0.05; Fig. 5a). Some of these genes were also up-regulated in E18.5 *Casp8*^*1xDA/1xDA*^ *Cflar*^*-/-*^ *Mlkl*^*-/-*^ intestines (for example, *Acod1*, *Ccl5*, *Cxcl10*, *Marco*, *Mmp9*, and *Nfkbia*). *Ccl5* and *Cxcl10* expression in *Casp8*^*1xDA/1xDA*^ *Cflar*^*-/-*^ *Mlkl*^*-/-*^ MEFs, like N4BP1 cleavage, was suppressed by *Fadd* or *Ripk1* deletion (Fig. 5b). *Ccl5* and *Cxcl10* expression was also reduced by deletion of the NF-κB transcription factor gene *RelA* (Fig. 5b). *Fadd* deletion in *Mlkl*^*-/-*^ control MEFs slightly increased *Ccl5* and *Cxcl10* expression, perhaps reflecting spontaneous RIPK1-RIPK3 complex formation in *Fadd*^*-/-*^ *Mlkl*^*-/-*^ cells [55]. Given that N4BP1 limits the duration of inflammatory gene expression triggered by certain TLRs [56], it is tempting to speculate that caspase-8 D387A cleavage of N4BP1 contributed to chemokine expression in *Casp8*^*1xDA/1xDA*^ *Cflar*^*-/-*^ *Mlkl*^*-/-*^ MEFs. We explored reconstituting the *Casp8*^*1xDA/1xDA*^ *Cflar*^*-/-*^ *Mlkl*^*-/-*^ MEFs with a non-cleavable form of N4BP1, but failed to identify mutations in N4BP1 that completely prevented its cleavage. Therefore, the significance of N4BP1 cleavage in *Casp8*^*1xDA/1xDA*^ *Cflar*^*-/-*^ *Mlkl*^*-/-*^ cells remains unclear.

Given that *Ccl5*, *Mmp9*, and *Nfkbia* are NF-κB target genes [57–59] and caspase-8 is implicated in NF-κB activation by certain death receptors [28, 29], we looked for corroborating evidence of increased NF-κB signaling in *Casp8*^*1xDA/1xDA*^ *Cflar*^*-/-*^ *Mlkl*^*-/-*^ cells. Western blotting revealed that 6 out of 9 E18.5 *Casp8*^*1xDA/1xDA*^ *Cflar*^*-/-*^ *Mlkl*^*-/-*^ intestines contained more phospho-p65/RelA than their *Mlkl*^*-/-*^ counterparts (Fig. 5c and data not shown), while a more subtle increase in phospho-p65/RelA was observed in *Casp8*^*1xDA/1xDA*^ *Cflar*^*-/-*^ *Mlkl*^*-/-*^ MEFs (Fig. S6a) and in *Casp8*^*1xDA/1xDA*^ MEFs after CRISPR deletion of *Cflar* (Fig. S5e). Collectively, our data suggest that cFLIP loss promotes the assembly of a RIPK1-FADD-caspase-8 D387A complex that drives transcriptional changes through NF-κB activation (Fig. S6b).

### Perinatal Lethality in *Casp8*^*1xDA/1xDA*^*Cflar*^*-/-*^*Mlkl*^*-/-*^ mice requires FADD

**Fig. 6:**
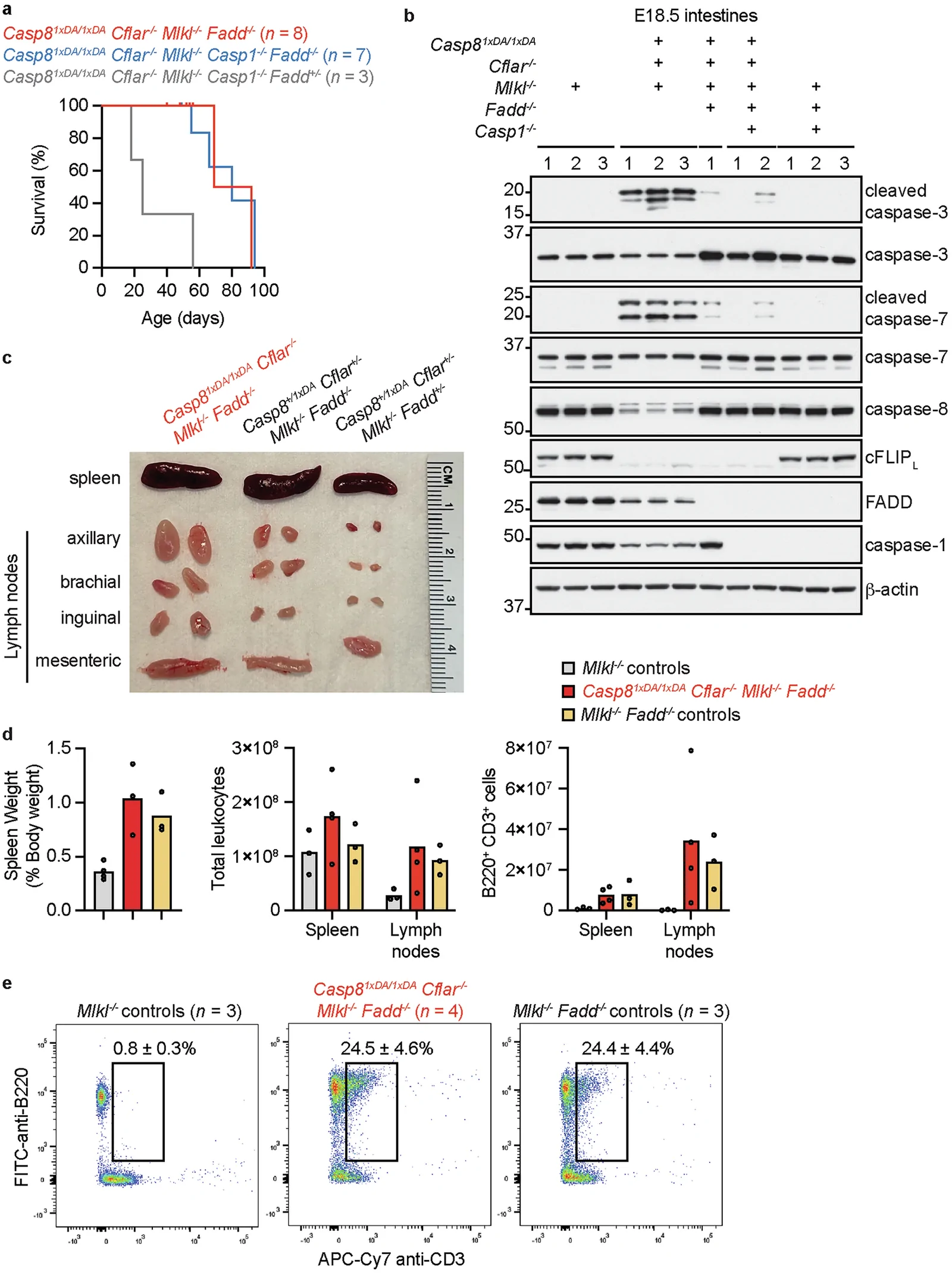
*Casp8*^*1xDA/1xDA*^*Cflar*^*-/-*^*Mlkl*^*-/-*^*Fadd*^*-/-*^ mice develop splenomegaly and lymphadenopathy. **a** Kaplan–Meier curves of mouse survival. **b** Western blots of E18.5 mouse intestines. Lanes, individual embryos. **c** Lymph nodes and spleens from mice aged 8 weeks. Results representative of 4 *Casp8*^*1xDA/1xDA*^ *Cflar*^*-/-*^ *Mlkl*^*-/-*^ *Fadd*^*-/-*^, 3 *Casp8*^*+/1xDA* or +/+^ *Cflar*^*+/-* or +/+^ *Mlkl*^*-/-*^ *Fadd*^*+/-*^, and 3 *Casp8*^*+/1xDA*^ *Cflar*^*+/-*^ *Casp1*^*+/-* or *+/+*^ *Mlkl*^*-/-*^ *Fadd*^*-/-*^ mice aged 7–8 weeks. **d** Spleen weights and leukocyte numbers (spleen or brachial, axillary, inguinal, and mesenteric lymph nodes) of the mice in (**c**). Bars indicate the mean. Circles, individual mice. **e** Representative dot plots of lymph node cells from the mice in (**c**). Percentages, mean ± s.e.m.

Next, we investigated whether FADD contributed to lethality in *Casp8*^*1xDA/1xDA*^ *Cflar*^*-/-*^ *Mlkl*^*-/-*^ mice. Both *Casp8*^*1xDA/1xDA*^ *Cflar*^*-/-*^ *Mlkl*^*-/-*^ *Fadd*^*-/-*^ and *Casp8*^*1xDA/1xDA*^ *Cflar*^*-/-*^ *Mlkl*^*-/-*^ *Fadd*^*-/-*^ *Casp1*^*-/-*^ mice were born at the expected frequency (Table 1) and survived beyond weaning (Fig. 6a), indicating that FADD loss is sufficient to rescue *Casp8*^*1xDA/1xDA*^ *Cflar*^*-/-*^ *Mlkl*^*-/-*^ perinatal lethality. Rescue coincided with reduced caspase-3/7 cleavage in the E18.5 intestine (Fig. 6b). Halving the dosage of *Fadd* yielded rare, undersized *Casp8*^*1xDA/1xDA*^ *Cflar*^*-/-*^ *Mlkl*^*-/-*^ *Fadd*^*+/-*^ *Casp1*^*-/-*^ mice (Table 1) with a median survival of 25 days (Fig. 6a). *Casp8*^*1xDA/1xDA*^ *Cflar*^*-/-*^ *Mlkl*^*-/-*^ *Fadd*^*-/-*^ and *Casp8*^*1xDA/1xDA*^ *Cflar*^*-/-*^ *Mlkl*^*-/-*^ *Fadd*^*-/-*^ *Casp1*^*-/-*^ mice were euthanized at 8–10 weeks of age because they, like *Mlkl*^*-/-*^ *Fadd*^*-/-*^ mice [25], developed lymphadenopathy and accumulated B220^+^ CD3^+^ T cells (Fig. 6c–e). Interestingly, E18.5 *Casp8*^*1xDA/1xDA*^ *Cflar*^*-/-*^ *Mlkl*^*-/-*^ *Fadd*^*-/-*^ *Casp1*^*-/-*^ intestines still exhibited dysregulated expression of many of the proinflammatory chemokines and cytokines observed in E18.5 *Casp8*^*1xDA/1xDA*^ *Cflar*^*-/-*^ *Mlkl*^*-/-*^ intestines, including *Cxcl10*, *Mx1*, and *Zbp1* (Fig. S7). This result argues that elevated expression of these proinflammatory genes is insufficient for perinatal lethality in *Casp8*^*1xDA/1xDA*^ *Cflar*^*-/-*^ *Mlkl*^*-/-*^ mice. Hence, the mechanism by which FADD and caspase-8 D387A trigger caspase-3 cleavage and intestinal atrophy remains ill-defined.

## Discussion

Previously, caspase-8 D387A was shown to exhibit proteolytic activity as heterodimers with cFLIP_L_, but not as homodimers [13]. Therefore, we and others speculated that the heterodimer protease permits sufficient extrinsic apoptosis to prevent LPR disease in *Casp8*^*1xDA/1xDA*^ and *Casp8*^*1xDA/1xDA*^ *Mlkl*^*-/-*^ mice [16, 40, 41]. Our experiments in MEFs support this model, because deleting all cFLIP isoforms in *Casp8*^*1xDA/1xDA*^ *Mlkl*^*-/-*^ cells rendered them completely resistant to TNF-induced apoptosis. A recent study showing that cFLIP loss causes LPR disease in homodimer-deficient *Casp8*^*FGLG/FGLG*^ *Mlkl*^*-/-*^ mice also concluded that heterodimers mediate FAS-induced apoptosis [60]. Collectively, these and other studies [43] indicate that either heterodimers or homodimers of caspase-8 can trigger extrinsic apoptosis.

In contrast to *Casp8*^*1xDA/1xDA*^ *Mlkl*^*-/-*^ and *Casp8*^*FGLG/FGLG*^ *Cflar*^*-/-*^ *Mlkl*^*-/-*^ mice [41], *Casp8*^*1xDA/1xDA*^ *Cflar*^*-/-*^ *Mlkl*^*-/-*^ mice did not survive the perinatal period. They resembled *Casp8*^*1xDA/1xDA*^ *Fadd*^*-/-*^ *Mlkl*^*-/-*^ [41], *Casp8*^*C362S/C362S*^ *Mlkl*^*-/-*^ [22], and *Casp8*^*C362A/C362A*^ *Mlkl*^*-/-*^ mice [47], demonstrating atrophy of the small intestine and markers of caspase-1 activation in the form of cleaved GSDMD, caspase-7, and caspase-3. However, *Casp8*^*1xDA/1xDA*^ *Cflar*^*-/-*^ *Mlkl*^*-/-*^ differed from these other genotypes as caspase-1 loss did not yield any survivors. A unique feature of *Casp8*^*1xDA/1xDA*^ *Cflar*^*-/-*^ *Mlkl*^*-/-*^ cells was low level cleavage of the caspase-8 substrates N4BP1 and RIPK1. Since these cleavage events were not detected in *Casp8*^*C362A/C362A*^ *Cflar*^*-/-*^ *Mlkl*^*-/-*^ cells, we inferred that they were mediated by caspase-8 D387A homodimers no longer held in check by cFLIP. The cFLIP antibody used in this study recognizes cFLIP_L_ and cFLIP_R_, but we have not detected cFLIP_R_ in MEFs, perhaps because it is relatively labile [15]. Abundant cFLIP_L_ will suppress rather than promote activation of caspase-8 [ref. 6], so cFLIP_L_ may be the isoform that limits activation of caspase-8 D387A. Whether TNF or another ligand drives basal caspase-8 D387A activation in cells lacking cFLIP is unclear. NOS2 has been implicated in caspase-8 activation in macrophages stimulated with IFNγ plus LPS [61], but whether the elevated *Nos2* expression observed in E18.5 *Casp8*^*1xDA/1xDA*^ *Cflar*^*-/-*^ *Mlkl*^*-/-*^ intestines is required for their atrophy is unclear. Efforts to rederive our colonies in a germ-free setting were unsuccessful, so it remains to be determined if the microbiome contributes to aberrant cell death in the intestines of *Casp8*^*C362A/C362A*^ *Mlkl*^*-/-*^ or *Casp8*^*1xDA/1xDA*^ *Cflar*^*-/-*^ *Mlkl*^*-/-*^ embryos.

Given that caspase-8 D387A homodimers could not cleave caspase-3 and trigger extrinsic apoptosis in *Casp8*^*1xDA/1xDA*^ *Cflar*^*-/-*^ *Mlkl*^*-/-*^ MEFs, it is unclear what drove caspase-3/7 cleavage and cell death in *Casp8*^*1xDA/1xDA*^ *Cflar*^*-/-*^ *Mlkl*^*-/-*^ *Casp1*^*-/-*^ intestines. We hypothesized that enhanced proinflammatory gene expression in the absence of cFLIP might promote cell death indirectly via the recruitment of immune cells. However, many proinflammatory transcripts were still elevated in the E18.5 intestines of “rescued” *Casp8*^*1xDA/1xDA*^ *Cflar*^*-/-*^ *Mlkl*^*-/-*^ *Fadd*^*-/-*^ *Casp1*^*-/-*^ mice. Proinflammatory gene expression without adverse consequences in the perinatal period has also been described in *Casp8*^*-/-*^ *Ripk3*^*-/-*^ embryos [62]. In this setting, proinflammatory gene expression was dependent on RIPK1. FADD deficiency prevented basal RIPK1 cleavage in *Casp8*^*1xDA/1xDA*^ *Cflar*^*-/-*^ *Mlkl*^*-/-*^ MEFs, so it is conceivable that RIPK1 also drives aberrant gene expression in E18.5 *Casp8*^*1xDA/1xDA*^ *Cflar*^*-/-*^ *Mlkl*^*-/-*^ *Fadd*^*-/-*^ *Casp1*^*-/-*^ intestines.

## Materials and methods

### Mice

*Fadd*^*+/-*^, *Casp8*^*+/C362A*^, *Casp8*^*1xDA*/*1xDA*^, *Casp8*^*4xDA*/*4xDA*^ [16], *Casp1*^*-/-*^ [63], *Ifnar1*^*-/-*^ [64], and *Mlkl*^*-/-*^ [65] mice had a C57BL/6N genetic background. Mice were designated E0.5 on the morning a vaginal plug was detected. Mice aged 6–14 weeks were dosed intravenously with 500 μg murine TNF (Genentech, South San Francisco, CA, USA) per kg body weight. Mice with severe lethargy or body temperature < 26 °C were euthanized. No statistical methods were used to determine group sizes. Studies were performed unblinded.

*Casp8*^*1xDA/1xDA*^ *Cflar*^*-/-*^ *Mlkl*^*-/-*^ mice were generated by electroporating *Casp8*^+/*1xDA*^ and *Casp8*^*1xDA*/*1xDA*^ zygotes with ribonucleoprotein (RNP) complexes of Cas9 (Integrated DNA Technologies, Coralville, IA, USA) and sgRNAs (Synthego, Redwood City, CA, USA) targeting *Cflar* (5′-ATGGCCATTGCTGCCGTGGG-3′ and 5′-TGGTGTCTGTGTTAACGTAG-3′) or *Mlkl* (5′-TCCACTCACAACCCATAGTC-3′ and 5′-GCTCGAGTTTGCATGTCGGA-3′). Zygotes were cultured overnight and embryo transfers performed at the 2-cell stage. A mosaic founder bearing a 3062 bp *Mlkl* deletion (GRCm38/mm10 chr8:111,330,941-111,334,002) and a 16,022 bp *Cflar* deletion (GRCm38/mm10 chr1:58,725,674-58,741,695) was selected for breeding.

*Casp8*^*C362A/C362A*^ *Cflar*^*-/-*^ *Mlkl*^*-/-*^ mice were generated by RNP microinjection of *Casp8*^*C362A/+*^ *Mlkl*^*-/*-^ zygotes [16]. Two sgRNAs targeting *Cflar* (5′-AACCTAGGGGTTTTTTCCG-3′ and 5′-CGAAAAGTTGATATAAGCC-3′) yielded a 788 bp *Cflar* deletion (GRCm38/mm10 chr1:58,728,611-58,729,398).

### Immunohistochemistry

Formalin-fixed, paraffin-embedded tissue sections were labeled with antibodies detecting cleaved caspase-3 [ref. 66], phospho-RIPK3 T231, S232 [ref. 67], or ZBP1 [ref. 68].

### Flow cytometry

Thymocytes, splenocytes, or lymph node cells (mesenteric, inguinal, axillary, and brachial) were labeled in 2% normal rat serum and 1 μg/mL 2.4G2 anti-CD16/CD32 with PE-conjugated GK1.5 anti-CD4, FITC-conjugated 53-6.7 anti-CD8, FITC-conjugated RA3-6B2 anti-B220 or APC-Cy7-conjugated 145-2C11 anti-CD3ε (BD Biosciences, Milpitas, CA, USA). Leukocytes were identified based on their forward scatter (FSC) and side scatter profiles. Dead cells stained by 7-AAD (BD Biosciences), plus doublets, identified by their FSC-A versus FSC-W profiles, were excluded from analyses. Data were acquired using a BD FACSCantoII cytometer (BD Biosciences) and analyzed with FlowJo 10.10.0.

### Cells

MEFs were generated by trypsin digestion of E14.5 embryos (head and internal organs removed). Cells were cultured in the high glucose version of Dulbecco’s modified Eagle medium (DMEM) supplemented with 10% heat-inactivated fetal bovine serum, 2 mM glutamine, 10 mM HEPES (pH 7.2), 1× non-essential amino acids solution, 100 U/mL penicillin and 100 μg/mL streptomycin in dishes pre-coated with 0.1% gelatin in PBS for <5 passages. sgRNAs (Integrated DNA Technologies) targeted:

*Fadd*: 5′-CCTGTCGGGCAACGATCTGA-3′ and 5′-GGACCTGTTCACGGTGCTGC-3′

*Rela*: 5′-TGTATTTCACGGGACCAGGC-3′ and 5′-CGGAGGAGTCCGGAACACAA-3′

*Ripk1*: 5′- ACACGTCCGACTTCTCCGT-3′ and 5′-CAACTCACTCAGCGCGGTT-3′

*Cflar*: 5′- ACCTGGCTGCACCTAACGTC-3′ and 5′- GGCGGTTTGACCTTCTCAAG-3′

Non-targeting control (NTC) Alt-R crRNAs were annealed to tracrRNAs. gRNAs were complexed with Alt-R S.p. Cas9 Nuclease V3 (Integrated DNA Technologies) at a 1.2:1 molar ratio. RNPs were electroporated into 2.5 × 10^5^ cells using a 4D-nucleofector system (Primary Cell Buffer P4; program CZ-167, Lonza, Walkersville, MD, USA). Cells were cultured for 3–7 days before harvesting.

MEFs were stimulated with 100 ng/mL mouse TNF (Genentech), 2 μM BV6 (Sigma-Aldrich, St Louis, MO, USA), 20 μM Z-VAD-FMK (Promega, Madison, WI, USA), 20 μM Emricasan (MedChemExpress, Monmouth Junction, NJ, USA), 100 ng/mL SuperFasLigand (Enzo Life Sciences, Farmingdale, NY, USA), 100 ng/mL SuperKiller TRAIL (Enzo Life Sciences), 10 μM GSK′843 (MedChemExpress), and 5 μg/mL cycloheximide (Sigma-Aldrich). To determine cell viability, adherent MEFs were detached with trypsin, pooled with floating cells, and stained with 2.5 μg/mL propidium iodide (PI; BD Biosciences). We used a Dead Cell Apoptosis Kit (Invitrogen, Thermo Fisher Scientific, Waltham, MA, USA) for Annexin V staining in Fig. S4. PI-negative viable cells were identified in a FACSCanto II cytometer. In Fig. S5c, viability was determined using CellTiter-Glo (Promega).

### Western blots

Lysates were prepared in 20 mM Tris-HCl pH 7.5, 135 mM NaCl, 1.5 mM MgCl_2_, 1 mM EGTA, 1% Triton X-100, 10% glycerol, phosSTOP phosphatase inhibitor (Roche Diagnostics, Mannheim, Germany), and Halt protease inhibitor cocktail (Pierce Biotechnology, Rockford, IL, USA). Tissues were mechanically disrupted using a bead mill homogenizer (Omni International, Kennesaw, GA, USA) and insoluble material removed by centrifugation (20,000 × *g*). Antibodies were from Genentech (1.28E12 anti-FADD, 2.21H2 anti-cFLIP, GN20-13 anti-GSDMD, 1G6.1.4 anti-RIPK3, GEN135-35-9 anti-phospho-RIPK3 T^231^ S^232^, GEN175-DP-B1 anti-RIPK1 S^166^ T^169^, GN58.3 anti-ZBP1), Cell Signaling Technology (Danvers, MA, USA; 9662 anti-caspase-3, 9664 anti-cleaved caspase-3, 9491 anti-cleaved caspase-7, 9492 anti-caspase-7, 8592 anti-cleaved caspase-8, 56343 anti-cFLIP, 10137 anti-cleaved GSDMD, 3493 anti-RIPK1), Sigma-Aldrich (anti-MLKL MABC604), MP Biomedicals (Burlingame, CA, USA; 69100 anti-β-actin), Enzo Life Sciences (1G12 anti-caspase-8), and Abcam (Waltham, MA, USA; ab215191 anti-GSDME, ab222407 anti-cleaved GSDME, ab133610 anti-N4BP1). 1.28E12 anti-FADD (Genentech) was used for immunoprecipitations. Uncropped western blots are provided as supplementary data.

### Quantitative RT-PCR

MEF RNA was isolated using a RNeasy Mini Kit (Qiagen, Redwood City, CA, USA) and an on-column DNase digestion. Transcripts were assessed using a Taqman RNA-to-CT 1-Step kit on a StepOnePlus Real-Time PCR System (Applied Biosystems, Waltham, MA, USA). Gene expression assays (Invitrogen) detected *Gapdh* (Mm99999915_g1), *Cxcl10* (Mm00445235_m1), and *Ccl5* (Mm01302427_m1). Ct values were normalized to *Gapdh* using the relative standard curve method. Fold-change was determined relative to a control sample (*Mlkl*^*-/-*^ NTC).

### RNA Sequencing

Snap frozen E18.5 intestines were placed in RNAlater-ICE frozen tissue transition solution (Invitrogen) overnight at −20 °C and then processed using a RNeasy Plus Mini Kit (Qiagen) with on-column DNase digestion. RNA was checked using the Agilent 2100 Bioanalyzer (Agilent Technologies, Santa Clara, CA, USA) and total RNA quantified with a Quant-iT RiboGreen RNA Kit (Thermo Fisher Scientific) on a Victor X2 Multilabel Microplate Reader (PerkinElmer, Waltham, MA, USA). RNA quality was assessed using High Sensitivity RNA ScreenTape on TapeStation 4200 (Agilent Technologies). Sequencing libraries were generated using a Truseq Stranded mRNA kit (Illumina, San Diego, CA, USA) and 100–1000 ng total RNA. Libraries were quantified with a Quant-iT PicoGreen dsDNA Assay Kit (Thermo Fisher Scientific) on a Victor X2 Multilabel Microplate Reader (PerkinElmer). The average library size was determined using D1000 ScreenTape on TapeStation 4200 (Agilent Technologies). Libraries were pooled and sequenced on a NovaSeq X Plus (Illumina) to generate 30 million single-end 50 bp reads for each sample.

MEF RNA was isolated with a RNeasy Mini Kit (Qiagen) and an on-column DNase digestion. RNA samples in Fig. S7 were processed in the same way as the intestinal RNA samples described above. RNA samples in Fig. 5a were quantified with a Qubit RNA HS Assay Kit (Thermo Fisher Scientific) and assessed using RNA ScreenTape on a TapeStation 4200 (Agilent Technologies). Sequencing libraries were generated using the KAPA mRNA HyperPrep Kit (Roche) and 50–500 ng total RNA. Libraries were quantified with a Qubit dsDNA HS Assay Kit (Thermo Fisher Scientific). The average library size was determined using D1000 ScreenTape on a TapeStation 4200 (Agilent Technologies). Libraries were pooled and sequenced on a NovaSeq 6000 or NovaSeq X Plus (Illumina) to generate 30 million single-end 50 bp reads for each sample.

### RNAseq data analysis

GSNAP (version 2013-11-10) [69] was used to align raw FASTQ reads to the mouse reference genome (GRCm38/mm10) with the following parameters “-M 2 -n 10 -B 2 -i 1 -N 1 -w 200000 -E 1 –pairmax-rna=200000 –clip-overlap”. Only uniquely mapped reads were used for the downstream analysis. Limma (version 3.54.1) [70] R package was used for differential expression analysis. Heatmaps were plotted using R package ComplexHeatmaps (version 2.18.0) [71, 72].

### Statistics and general methods

Statistics were calculated in Prism (version 10.3.1). Where possible, we analyzed at least 3 samples or cells of each genotype. No samples or animals were excluded from analyses. Samples and animals were allocated to groups based on genotype without randomization. No blinding was done.

## Supplementary information

Figures S1 to S7

Uncropped western blots

## Data Availability

RNAseq data have been deposited to GEO (GSE299620). Unique reagents generated in this study may be requested through Genentech’s MTA program.
