# Supplementary material for: cFLIP suppresses caspase-1- and MLKL-independent perinatal lethality driven by auto-processing impaired caspase-8 D387A
Source: Cell Death Differ. 2025 Dec 12;33(9):1745–55. doi: 10.1038/s41418-025-01650-0 (PMC13545278; doi:10.1038/s41418-025-01650-0)
Supplement: Supplementary file 1 [file 41418_2025_1650_MOESM1_ESM.pdf]

Supplementary Figure 1. Characterization of *Casp8*$^{1xDA/1xDA}$ *Cflar*$^{-/-}$ *Ripk1*$^{D138N/D138N}$ and *Casp8*$^{1xDA/1xDA}$ *Cflar*$^{-/-}$ *Mlkl*$^{-/-}$ mice.

(**a**) E12.5 littermates. Results representative of 5 *Casp8*$^{1xDA/1xDA}$ *Cflar*$^{-/-}$ *Ripk1*$^{D138N/D138N}$ and 4 *Ripk1*$^{D138N/D138N}$ embryos.

(**b**) E18.5 thymocyte numbers. Lines indicate the mean. Circles, individual embryos (*Mlkl*$^{-/-}$ n=7; *Casp8*$^{1xDA/1xDA}$ *Cflar*$^{-/-}$ *Mlkl*$^{-/-}$ n=4).

(**c**) Representative dot plots of the thymocytes in (b). Percentages, mean ± s.e.m.

(**d**) Concentrations of IL-6 and CXCL9 in E18.5 intestines. Bars indicate the mean. Circles, individual embryos (*Mlkl*$^{-/-}$ n=2 or 3; *Casp8*$^{1xDA/1xDA}$ *Cflar*$^{-/-}$ *Mlkl*$^{-/-}$ n=3).

(**e**) Volcano plot indicating differentially expressed genes in E18.5 *Casp8*$^{1xDA/1xDA}$ intestines (n=3) compared with *Mlkl*$^{-/-}$ intestines (n=3). FC, fold change. FDR, false discovery rate. Blue dots, downregulated genes. Red dots, upregulated genes.

(**f**) 4-way plot comparing differentially expressed genes in E18.5 *Casp8*$^{1xDA/1xDA}$ *Cflar*$^{-/-}$ *Mlkl*$^{-/-}$ or *Casp8*$^{C362A/C362A}$ *Cflar*$^{-/-}$ *Mlkl*$^{-/-}$ intestines when compared with *Mlkl*$^{-/-}$ littermate intestines (n=3 per genotype). Red dots, genes dysregulated in both comparisons (FDR < 0.05, Log2 FC > 1 or < -1). Green dots, genes dysregulated only *Casp8*$^{C362A/C362A}$ *Cflar*$^{-/-}$ *Mlkl*$^{-/-}$ intestines (FDR < 0.05, Log2 FC > 1 or < -1 on x-axis, and FDR ≥ 0.05 on y-axis). Blue dots, genes dysregulated only in *Casp8*$^{1xDA/1xDA}$ *Cflar*$^{-/-}$ *Mlkl*$^{-/-}$ intestines (FDR < 0.05, Log2 FC > 1 or < -1 on y-axis, and FDR $\geq$ 0.05 on x-axis).

(**g**) Heatmaps indicate the expression of genes upregulated (FDR < 0.05, Log2 FC > 1) in E18.5 *Casp8*$^{1xDA/1xDA}$ *Cflar*$^{-/-}$ *Mlkl*$^{-/-}$ and *Casp8*$^{C362A/C362A}$ *Mlkl*$^{-/-}$ intestines when compared with *Mlkl*$^{-/-}$ littermate intestines (n=3 per genotype).

(**h**) E18.5 intestine sections immunolabelled for cleaved caspase-3. Scale bar, 100 µm. Results representative of 2 *Mlkl*$^{-/-}$ and 2 *Casp8*$^{1xDA/1xDA}$ *Cflar*$^{-/-}$ *Mlkl*$^{-/}$ embryos.

Supplementary Figure 2. Loss of cFLIP reduces detergent-soluble caspase-8 in the intestine.

Western blots of E18.5 intestines (n=2 per genotype).

Supplementary Figure 3. Caspase-8 D387A skews TNF-induced cell death signaling in favor of necroptosis.

(**a**) Western blots of MEFs treated with 100 ng/mL TNF and 5 µg/mL cycloheximide (T+C). Results representative of 2 independent experiments.

(**b**) Percentage of surviving MEFs at 16 h after treatment. Bars indicate the mean. Circles, cells from individual embryos (n=3 per genotype).

(**c**) Graphs indicate body temperatures (mean ± s.e.m.) and survival of mice dosed with 500 µg TNF per kg body weight. M, males. F, females. *P*-values (survival) <0.05 by two-sided Log-rank test are shown.

Supplementary Figure 4. The combination of TNF, BV6, plus GSK′843 induces apoptosis in MEFs.

(**a, b**) Flow cytometry analysis of MEFs stained with PI and Annexin V. Percentages are the mean ± s.e.m. of MEFs from 3 independent embryos.

Supplementary Figure 5. Characterization of *Gsdmd*$^{-/-}$ *Gsdme*$^{-/-}$ and *Casp8*$^{1xDA/1xDA}$ *Cflar*$^{-/-}$ *Mlkl*$^{-/-}$ MEFs.

(**a, d-g**) Western blots of MEFs. Lanes in (d-g), MEFs from individual embryos. MEFs from embryo #7 (d) are also shown in Fig. 4c.

(**b, c**) Percentage of viable MEFs by Cell Titer Glo assay at 16 h after treatment (b) or at the times indicated (c). Bars indicate the mean. Circles, cells from individual embryos (n=3 per genotype).

Supplementary Figure 6. *Casp8*$^{1xDA/1xDA}$ *Cflar*$^{-/-}$ *Mlkl*$^{-/-}$ MEFs exhibit a subtle increase in p65/RelA phosphorylation.

(**a**) Western blots of MEFs. Lanes, MEFs from individual embryos.

(**b**) Model for the signaling pathways perturbed by impaired caspase-8 autoprocessing with and without cFLIP loss. In most WT cells, caspase-8 signaling complexes are transient owing to caspase-8 cleaving itself, cFLIP and RIPK1. However, if cFLIP is low, then robust cleavage of caspase-3 elicits apoptosis. Caspase-8 D387A/FLIP heterodimers favor necroptosis rather than apoptosis, attributable to less robust cleavage of RIPK1 and caspase-3. When cFLIP is eliminated, caspase-8 D387A promotes necroptosis, as seen in E10.5 embryos. When cFLIP and MLKL are eliminated, the RIPK1-FADD-caspase-8 D387A complex drives proinflammatory gene expression and pyroptosis induction, as seen in newborn mice. The induction of death ligands may form a feed forward loop for sustained caspase-8 D387A complex assembly, such that low level cleavage of the caspase-8 substrates RIPK1 and N4BP1 is detected. Apoptosis in *Casp8*$^{1xDA/1xDA}$ *Cflar*$^{-/-}$ *Mlkl*$^{-/-}$ intestines may be secondary to inflammation or there may be caspase-3 cleavage by caspase-8 D387A that is not detected in MEFs. The contribution of aberrant N4BP1 cleavage to the phenotype of *Casp8*$^{1xDA/1xDA}$ *Cflar*$^{-/-}$ *Mlkl*$^{-/-}$ mice is unclear.

Supplementary Figure 7. Characterization of *Casp8$^{1xDA/1xDA}$ Cflar$^{-/-}$ Mlkl$^{-/-}$ Fadd$^{-/-}$ Casp1$^{-/-}$* embryos.

(**a**) 4-way plot comparing differentially expressed genes in E18.5 *Casp8$^{1xDA/1xDA}$ Cflar$^{-/-}$ Mlkl$^{-/-}$* or *Casp8$^{1xDA/1xDA}$ Cflar$^{-/-}$ Mlkl$^{-/-}$ Casp1$^{-/-}$ Fadd$^{-/-}$* intestines when compared with *Mlkl$^{-/-}$* intestines (n=3 per genotype). Red dots, genes dysregulated in both comparisons (FDR < 0.05, Log2 FC > 1 or < -1). Green dots, genes dysregulated only in *Casp8$^{1xDA/1xDA}$ Cflar$^{-/-}$ Mlkl$^{-/-}$ Casp1$^{-/-}$ Fadd$^{-/-}$* intestines (FDR < 0.05, Log2 FC > 1 or < -1 on y-axis, and FDR ≥ 0.05 on x-axis). Blue dots, genes dysregulated only in *Casp8$^{1xDA/1xDA}$ Cflar$^{-/-}$ Mlkl$^{-/-}$* intestines (FDR < 0.05, Log2 FC > 1 or < -1 on x-axis, and FDR ≥ 0.05 on y-axis).

(**b**) Transcript levels in E18.5 intestines. Bars indicate the mean (n=3 per genotype). Circles, individual embryos.

Figure S1

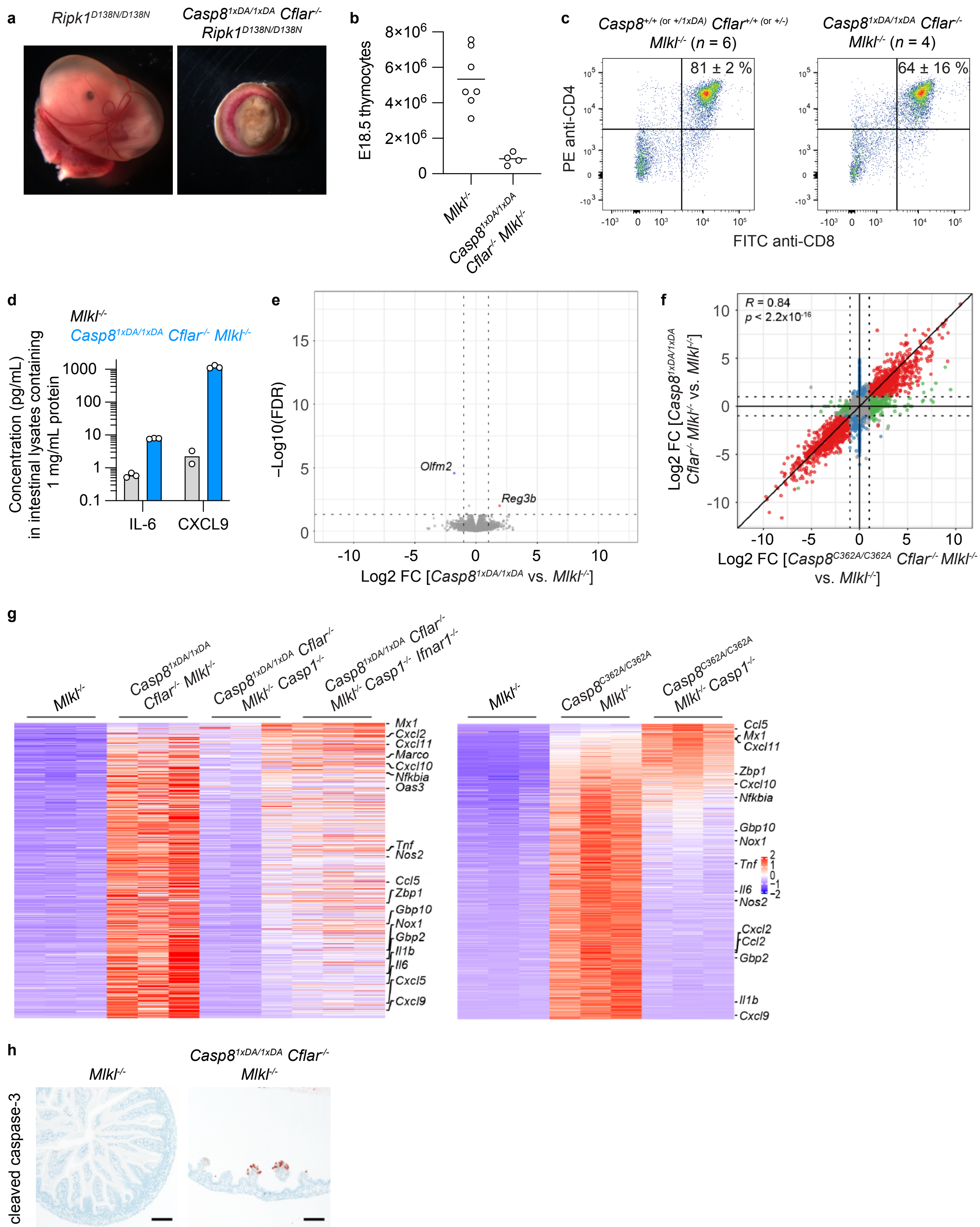
a
Ripk1D138N/D138N
Casp81xDA/1xDA Cflar-/- Ripk1D138N/D138N
b
E18.5 thymocytes
8×10^6
6×10^6
4×10^6
2×10^6
0
Mlkl-/-
Casp81xDA/1xDA Cflar-/- Mlkl-/-
c
Casp8+/+ (or +/1xDA) Cflar+/+ (or +/-) Mlkl-/- (n = 6)
Casp81xDA/1xDA Cflar-/- Mlkl-/- (n = 4)
81 ± 2 %
64 ± 16 %
PE anti-CD4
FITC anti-CD8
d
Mlkl-/-
Casp81xDA/1xDA Cflar-/- Mlkl-/-
Concentration (pg/mL) in intestinal lysates containing 1 mg/mL protein
1000
100
10
1
0.1
IL-6
CXCL9
e
−Log10(FDR)
Olfm2
Reg3b
Log2 FC [Casp81xDA/1xDA vs. Mlkl-/-]
f
R = 0.84
p < 2.2x10-16
Log2 FC [Casp81xDA/1xDA Cflar-/- Mlkl-/- vs. Mlkl-/-]
Log2 FC [Casp8C362A/C362A Cflar-/- Mlkl-/- vs. Mlkl-/-]
g
Mlkl-/-
Casp81xDA/1xDA Cflar-/- Mlkl-/-
Casp81xDA/1xDA Cflar-/- Mlkl-/- Casp1-/-
Casp81xDA/1xDA Cflar-/- Mlkl-/- Casp1-/- Ifnar1-/-
Mx1
Cxcl2
Cxcl11
Marco
Cxcl10
Nfkbia
Oas3
Tnf
Nos2
Ccl5
Zbp1
Gbp10
Nox1
Gbp2
Il1b
Il6
Cxcl5
Cxcl9
Mlkl-/-
Casp8C362A/C362A Mlkl-/-
Casp8C362A/C362A Mlkl-/- Casp1-/-
Ccl5
Mx1
Cxcl11
Zbp1
Cxcl10
Nfkbia
Gbp10
Nox1
Tnf
Il6
Nos2
Cxcl2
Ccl2
Gbp2
Il1b
Cxcl9
2
1
0
-1
-2
h
Mlkl-/-
Casp81xDA/1xDA Cflar-/- Mlkl-/-
cleaved caspase-3

Figure S2

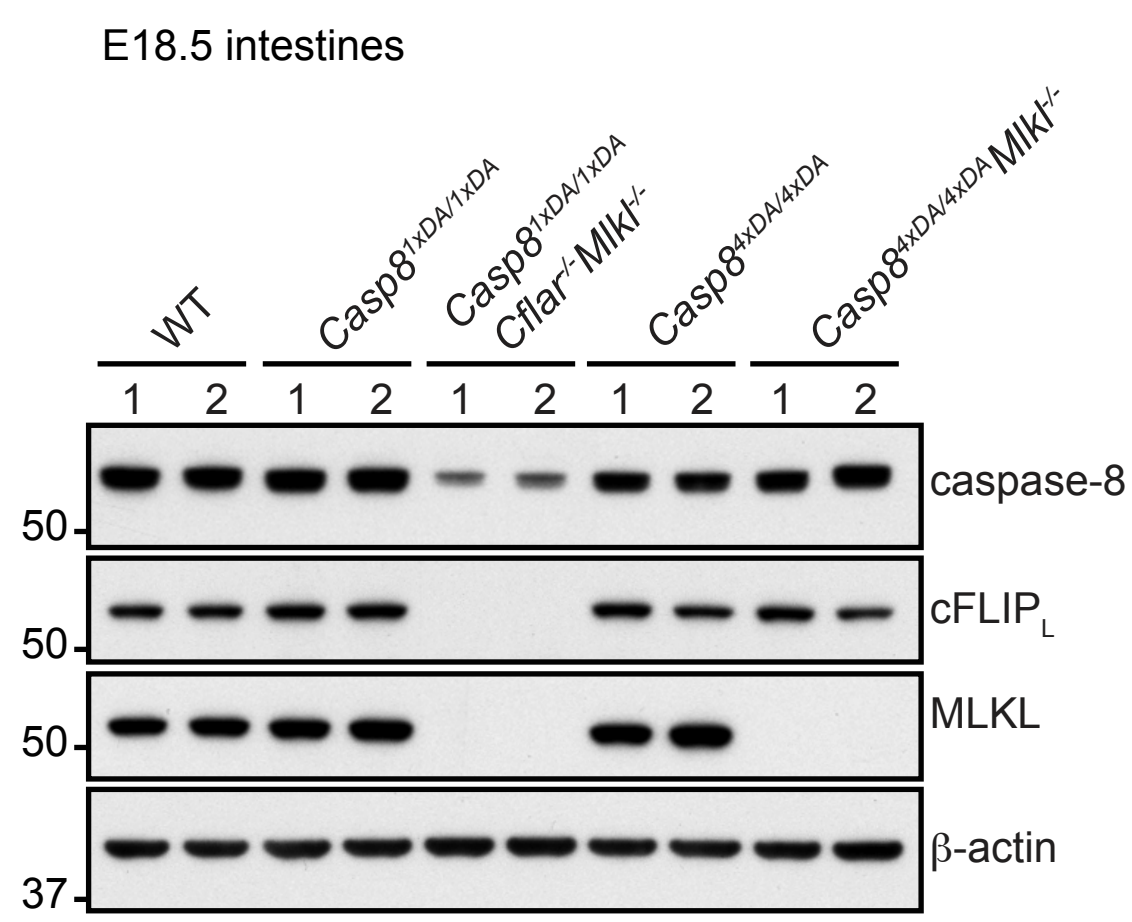
E18.5 intestines
WT
$Casp8^{1xDA/1xDA}$
$Casp8^{1xDA/1xDA}$ $Cflar^{-/-}Mlkl^{-/-}$
$Casp8^{4xDA/4xDA}$
$Casp8^{4xDA/4xDA}Mlkl^{-/-}$
1 2 1 2 1 2 1 2 1 2
caspase-8
$cFLIP_L$
MLKL
β-actin
50
50
50
37

Figure S3

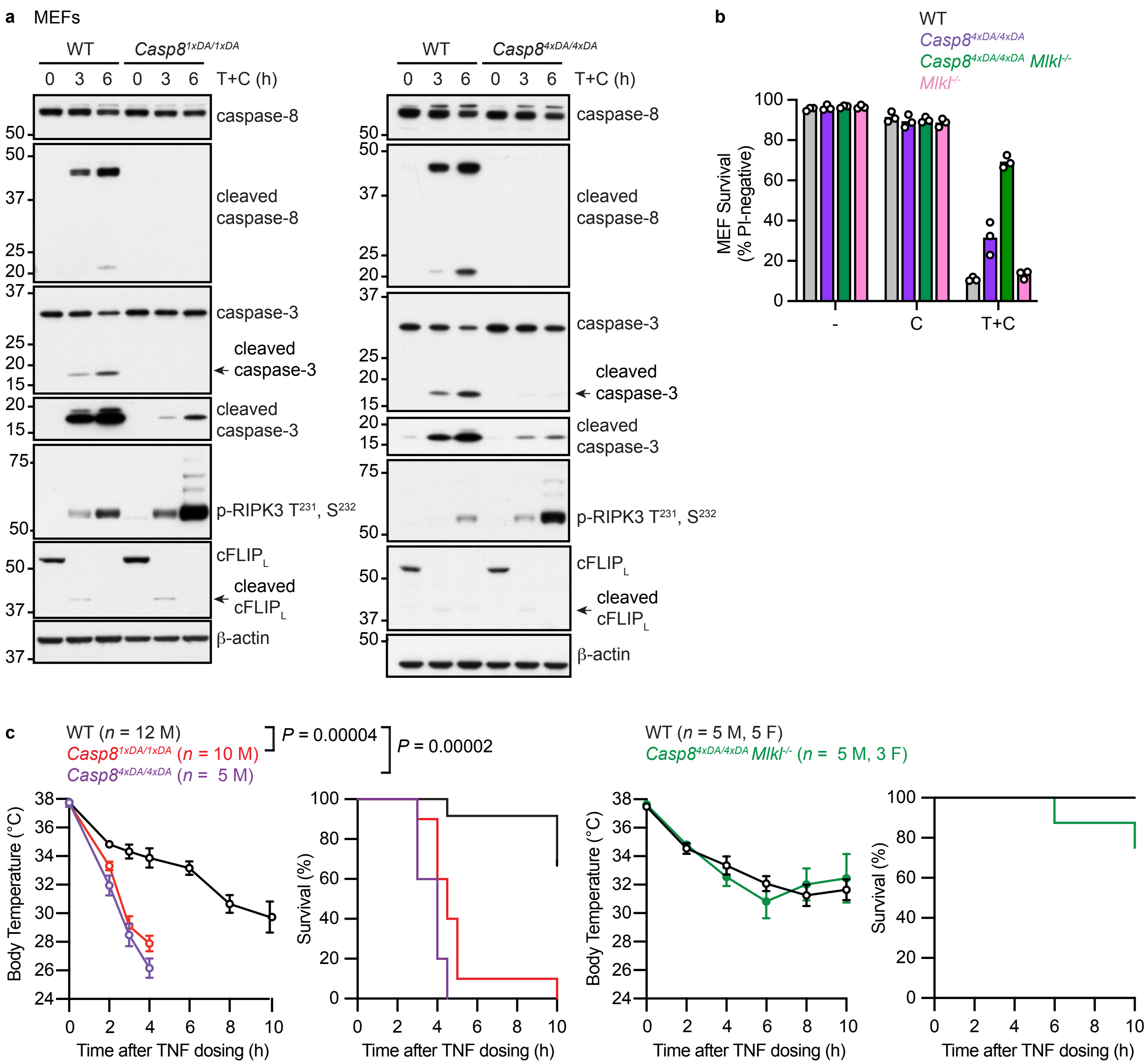

a
MEFs
WT
Casp8 1xDA/1xDA
0 3 6 0 3 6 T+C (h)
caspase-8
cleaved caspase-8
caspase-3
cleaved caspase-3
cleaved caspase-3
p-RIPK3 T231, S232
cFLIPL
cleaved cFLIPL
β-actin
WT
Casp8 4xDA/4xDA
0 3 6 0 3 6 T+C (h)
b
WT
Casp8 4xDA/4xDA
Casp8 4xDA/4xDA Mlkl-/-
Mlkl-/-
MEF Survival (% PI-negative)
-
C
T+C
c
WT (n = 12 M)
Casp8 1xDA/1xDA (n = 10 M)
Casp8 4xDA/4xDA (n = 5 M)
P = 0.00004
P = 0.00002
WT (n = 5 M, 5 F)
Casp8 4xDA/4xDA Mlkl-/- (n = 5 M, 3 F)
Body Temperature (°C)
Survival (%)
Time after TNF dosing (h)

Figure S4

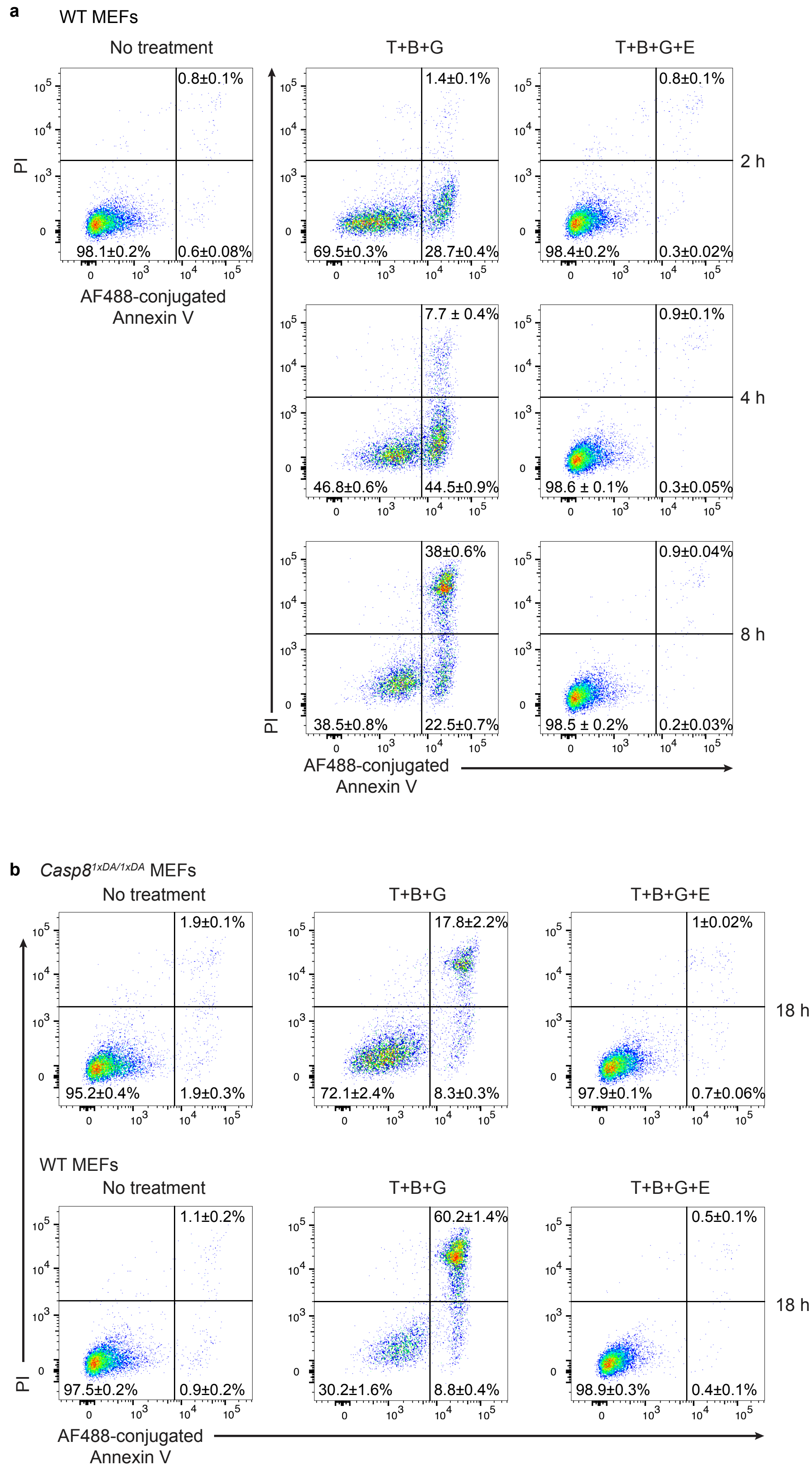
a
WT MEFs
No treatment
T+B+G
T+B+G+E
PI
AF488-conjugated Annexin V
2 h
4 h
8 h
0.8±0.1%
98.1±0.2%
0.6±0.08%
1.4±0.1%
69.5±0.3%
28.7±0.4%
0.8±0.1%
98.4±0.2%
0.3±0.02%
7.7 ± 0.4%
46.8±0.6%
44.5±0.9%
0.9±0.1%
98.6 ± 0.1%
0.3±0.05%
38±0.6%
38.5±0.8%
22.5±0.7%
0.9±0.04%
98.5 ± 0.2%
0.2±0.03%
b
Casp8 1xDA/1xDA MEFs
No treatment
T+B+G
T+B+G+E
18 h
1.9±0.1%
95.2±0.4%
1.9±0.3%
17.8±2.2%
72.1±2.4%
8.3±0.3%
1±0.02%
97.9±0.1%
0.7±0.06%
WT MEFs
No treatment
T+B+G
T+B+G+E
18 h
1.1±0.2%
97.5±0.2%
0.9±0.2%
60.2±1.4%
30.2±1.6%
8.8±0.4%
0.5±0.1%
98.9±0.3%
0.4±0.1%
PI
AF488-conjugated Annexin V

Figure S5

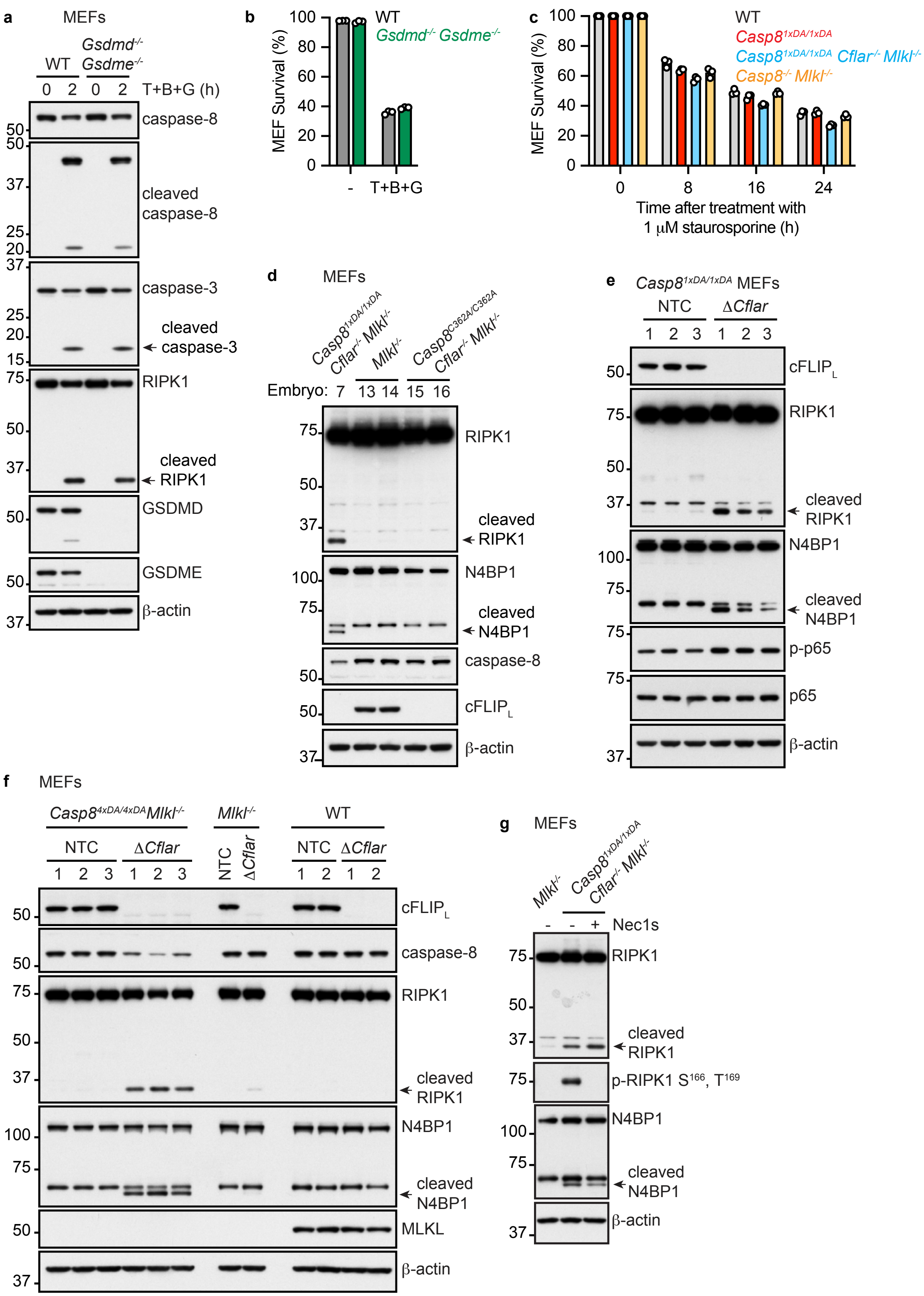
a
MEFs
WT
Gsdmd-/- Gsdme-/-
0 2 0 2 T+B+G (h)
caspase-8
cleaved caspase-8
caspase-3
cleaved caspase-3
RIPK1
cleaved RIPK1
GSDMD
GSDME
β-actin
b
MEF Survival (%)
WT
Gsdmd-/- Gsdme-/-
-
T+B+G
c
MEF Survival (%)
WT
Casp8 1xDA/1xDA
Casp8 1xDA/1xDA Cflar-/- Mlkl-/-
Casp8-/- Mlkl-/-
Time after treatment with 1 μM staurosporine (h)
d
MEFs
Casp8 1xDA/1xDA Cflar-/- Mlkl-/-
Mlkl-/-
Casp8 C362A/C362A Cflar-/- Mlkl-/-
Embryo: 7 13 14 15 16
RIPK1
cleaved RIPK1
N4BP1
cleaved N4BP1
caspase-8
cFLIP L
β-actin
e
Casp8 1xDA/1xDA MEFs
NTC
ΔCflar
cFLIP L
RIPK1
cleaved RIPK1
N4BP1
cleaved N4BP1
p-p65
p65
β-actin
f
MEFs
Casp8 4xDA/4xDA Mlkl-/-
Mlkl-/-
WT
NTC
ΔCflar
cFLIP L
caspase-8
RIPK1
cleaved RIPK1
N4BP1
cleaved N4BP1
MLKL
β-actin
g
MEFs
Mlkl-/-
Casp8 1xDA/1xDA Cflar-/- Mlkl-/-
- - + Nec1s
RIPK1
cleaved RIPK1
p-RIPK1 S166, T169
N4BP1
cleaved N4BP1
β-actin

Figure S6

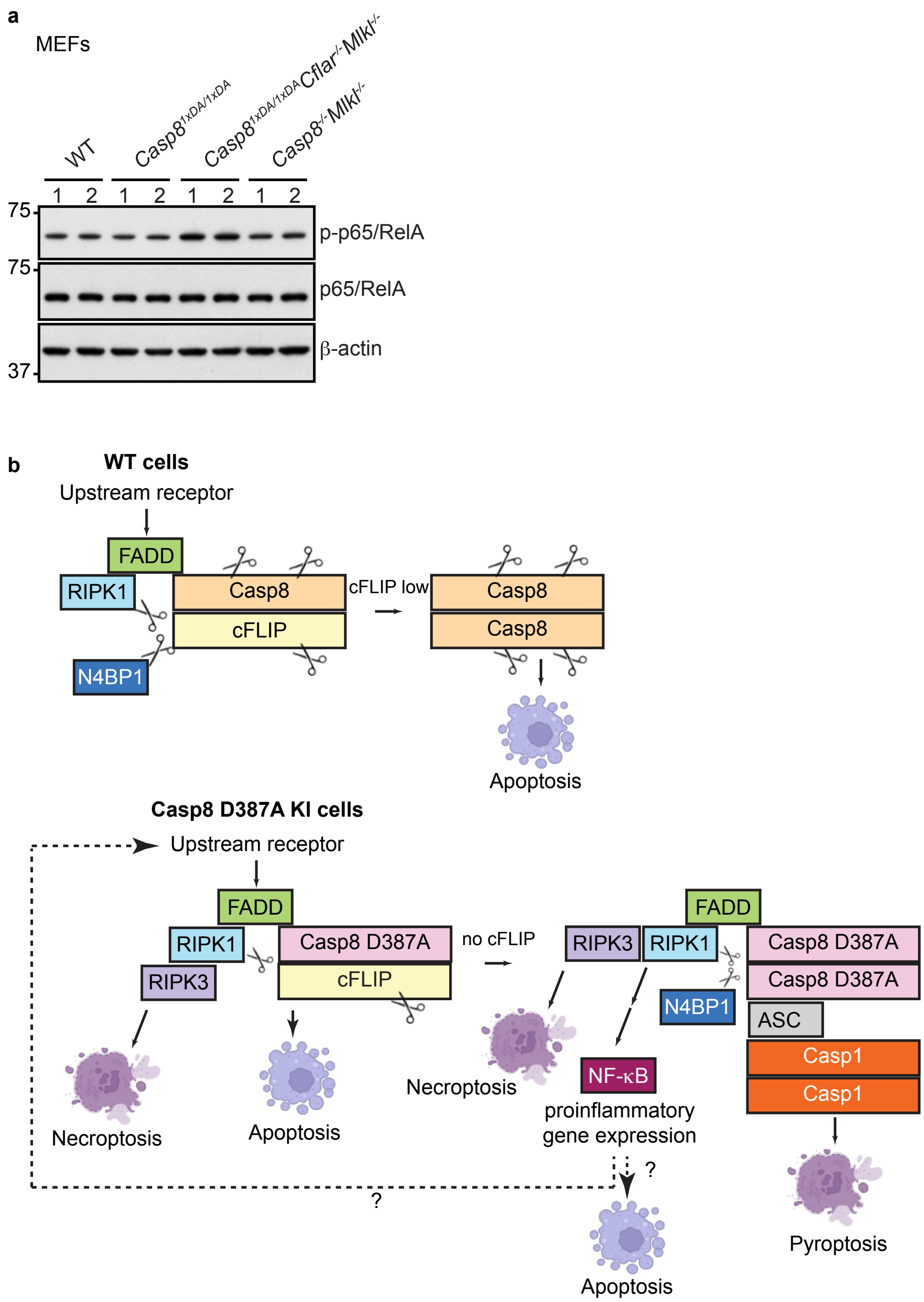

a
MEFs
WT
Casp8^1xDA/1xDA
Casp8^1xDA/1xDA Cflar-/- Mlkl-/-
Casp8-/- Mlkl-/-
1 2 1 2 1 2 1 2
75
p-p65/RelA
75
p65/RelA
β-actin
37
b
WT cells
Upstream receptor
FADD
RIPK1
Casp8
cFLIP
N4BP1
cFLIP low
Casp8
Casp8
Apoptosis
Casp8 D387A KI cells
Upstream receptor
FADD
RIPK1
RIPK3
Casp8 D387A
cFLIP
Necroptosis
Apoptosis
no cFLIP
FADD
RIPK3
RIPK1
Casp8 D387A
Casp8 D387A
N4BP1
ASC
Casp1
Casp1
Necroptosis
NF-κB
proinflammatory gene expression
?
?
Apoptosis
Pyroptosis

Figure S7

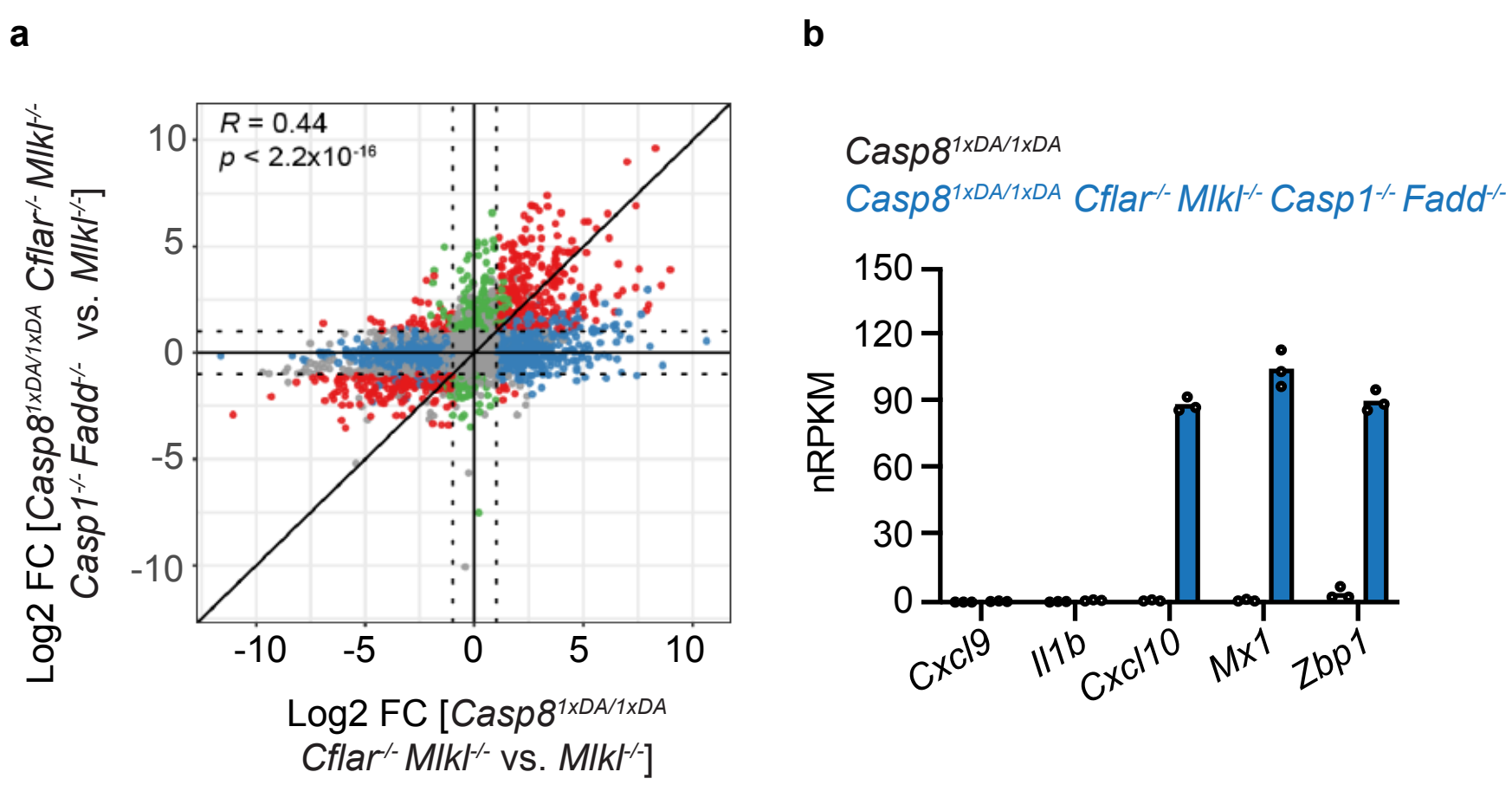
a
R = 0.44
p < 2.2x10-16
10
5
0
-5
-10
Log2 FC [Casp8 1xDA/1xDA Cflar-/- Mlkl-/- Casp1-/- Fadd-/- vs. Mlkl-/-]
Log2 FC [Casp8 1xDA/1xDA Cflar-/- Mlkl-/- vs. Mlkl-/-]
b
Casp8 1xDA/1xDA
Casp8 1xDA/1xDA Cflar-/- Mlkl-/- Casp1-/- Fadd-/-
150
120
90
60
30
nRPKM
Cxcl9
Il1b
Cxcl10
Mx1
Zbp1
