# Supplementary material for: cFLIP suppresses caspase-1- and MLKL-independent perinatal lethality driven by auto-processing impaired caspase-8 D387A
Source: Cell Death Differ. 2025 Dec 12;33(9):1745–55. doi: 10.1038/s41418-025-01650-0 (PMC13545278; doi:10.1038/s41418-025-01650-0)

Figure 3a

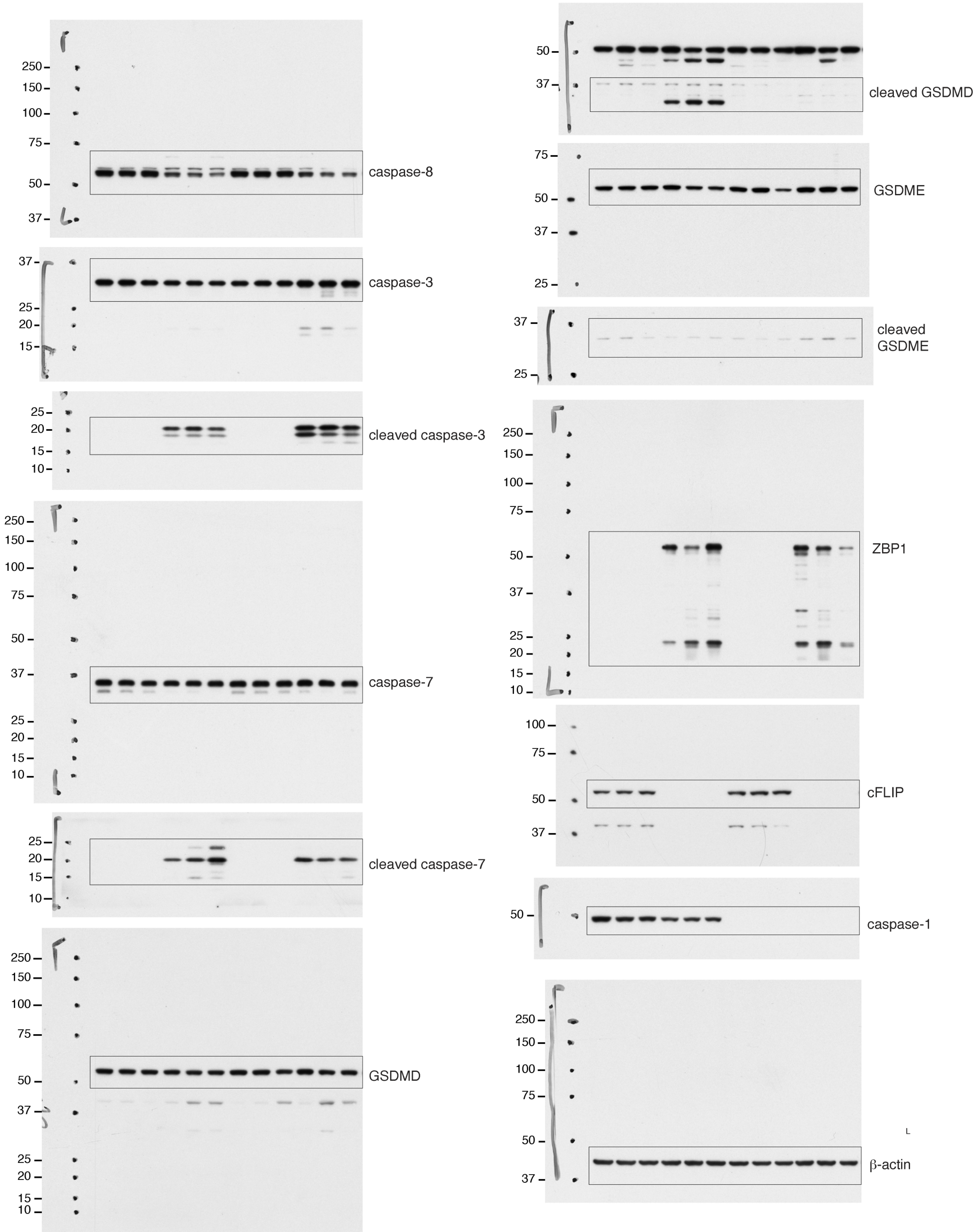

250
150
100
75
50
37
caspase-8
37
25
20
15
caspase-3
25
20
15
10
cleaved caspase-3
250
150
100
75
50
37
25
20
15
10
caspase-7
25
20
15
10
cleaved caspase-7
250
150
100
75
50
37
25
20
15
10
GSDMD
50
37
cleaved GSDMD
75
50
37
25
GSDME
37
25
cleaved
GSDME
250
150
100
75
50
37
25
20
15
10
ZBP1
100
75
50
37
cFLIP
50
caspase-1
250
150
100
75
50
37
β-actin

Figure 4b

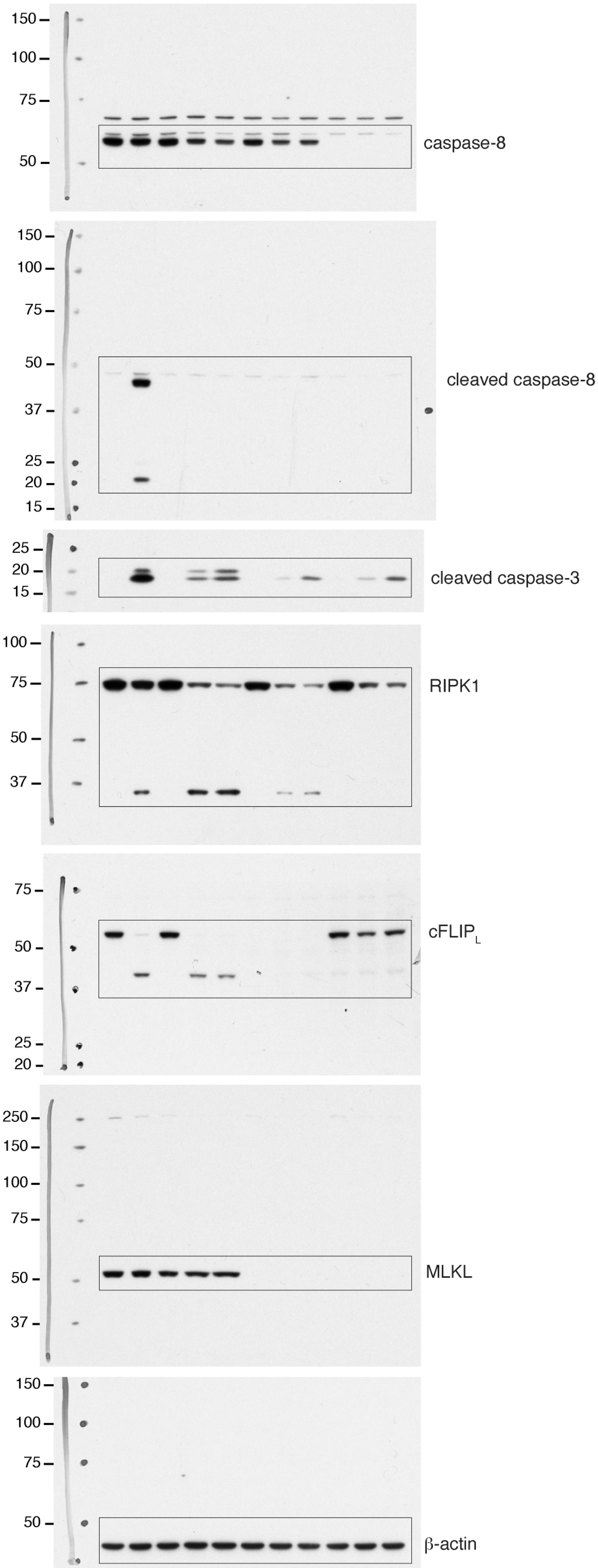

Figure 4c

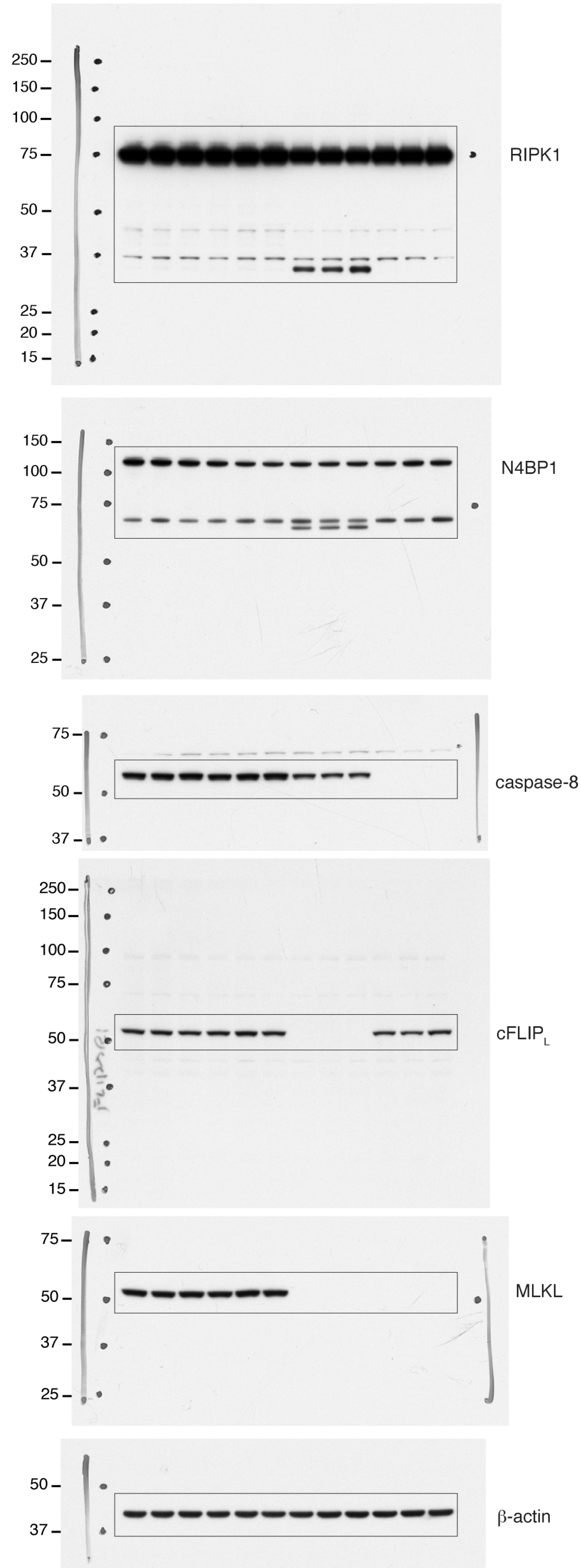

Figure 4d

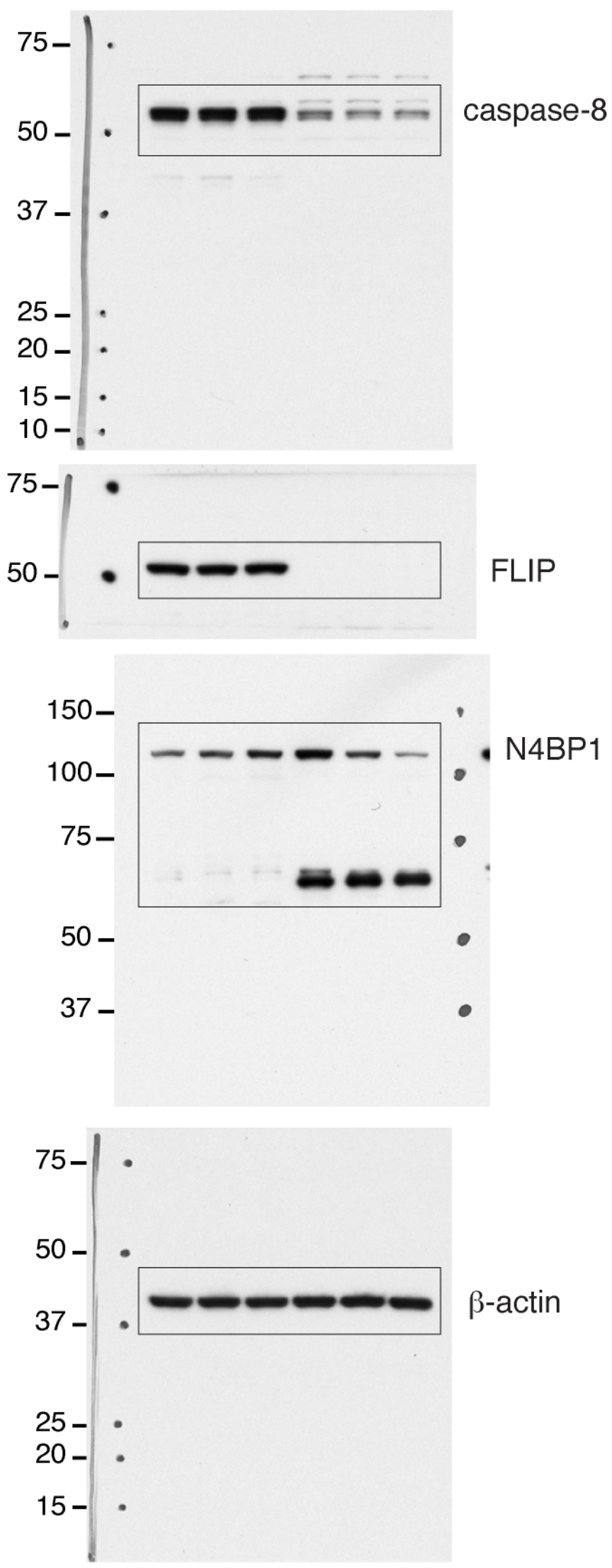

Figure 4e

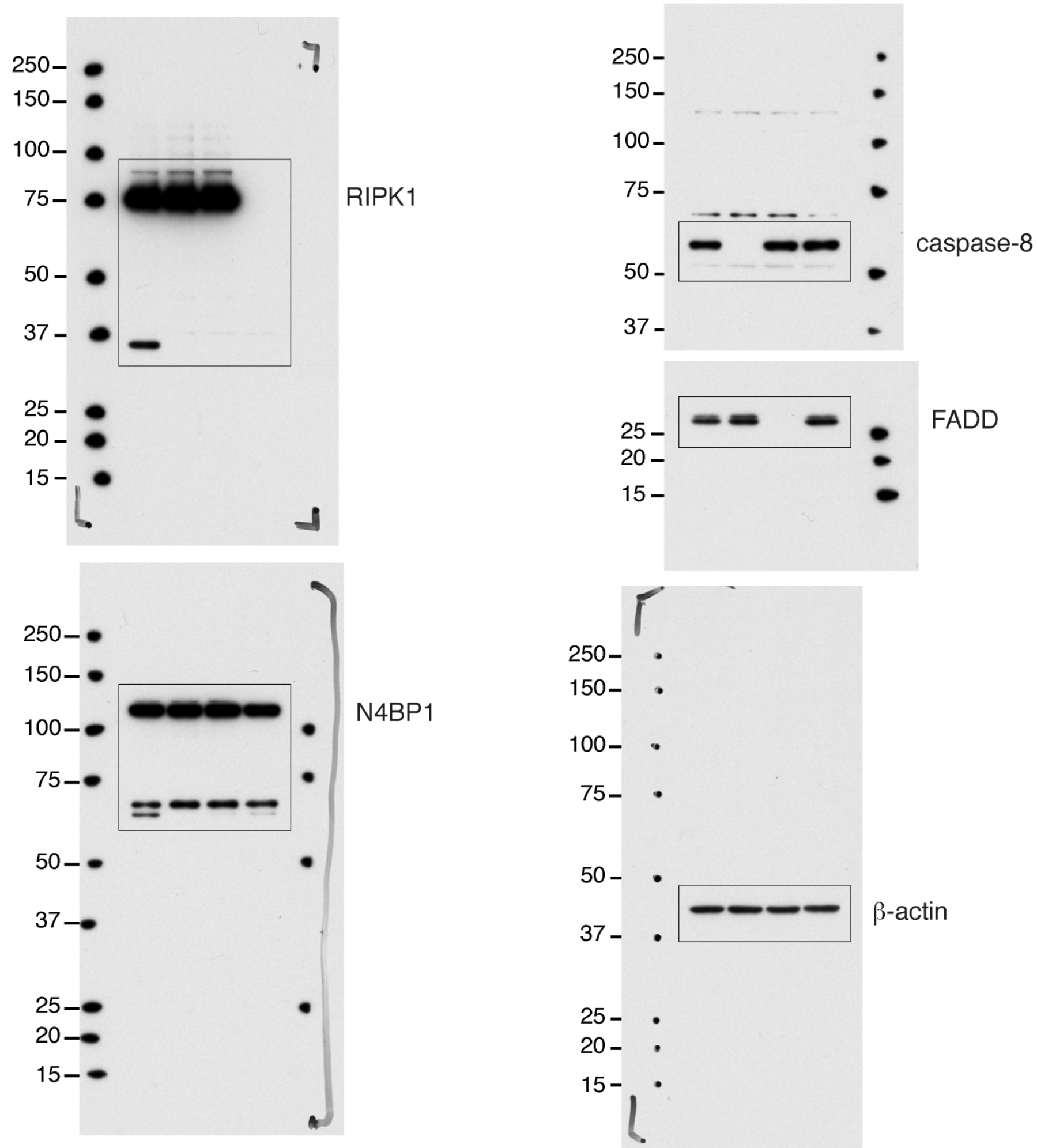

Figure 4f

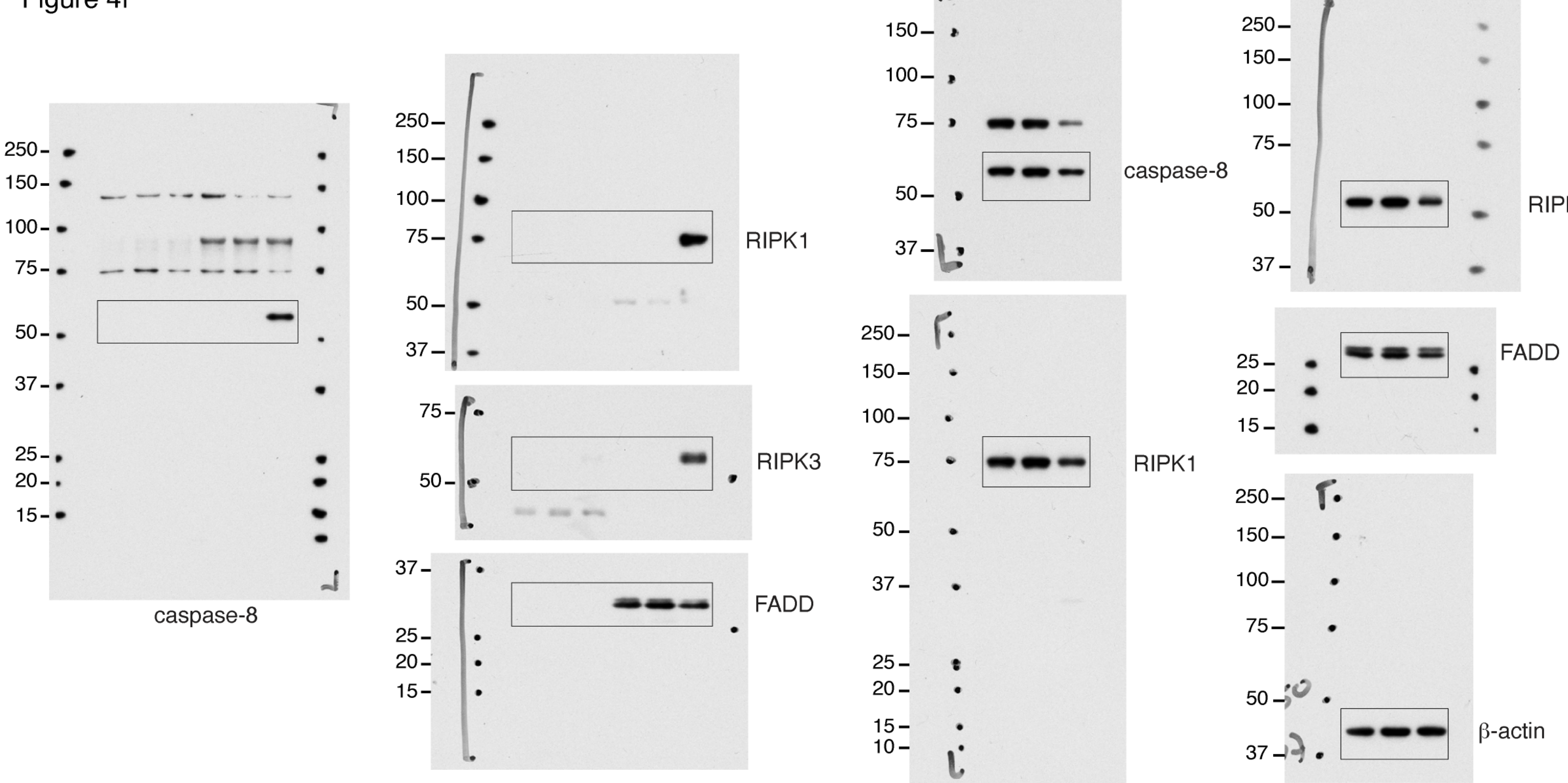

Figure 5b

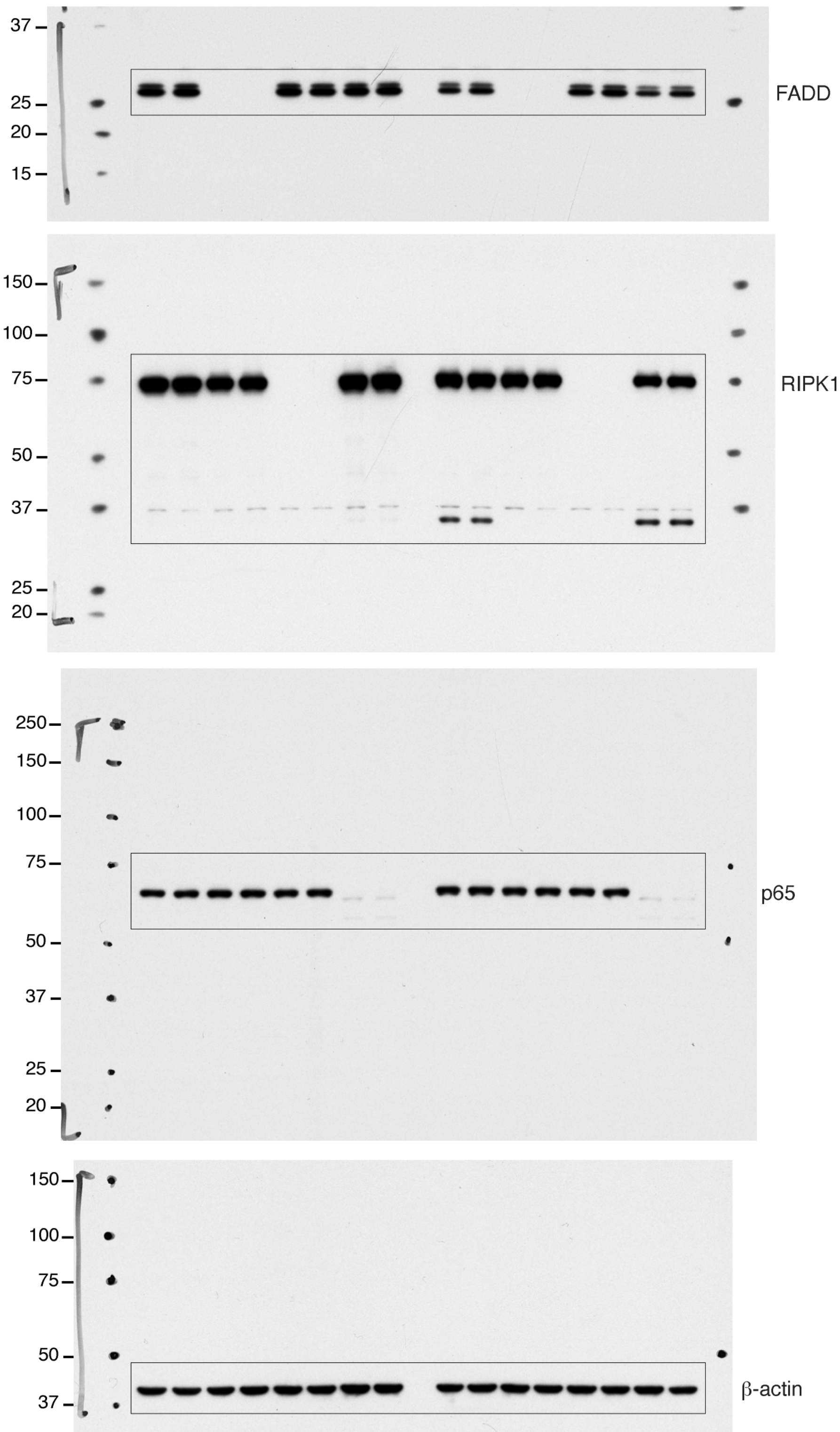

Figure 5c

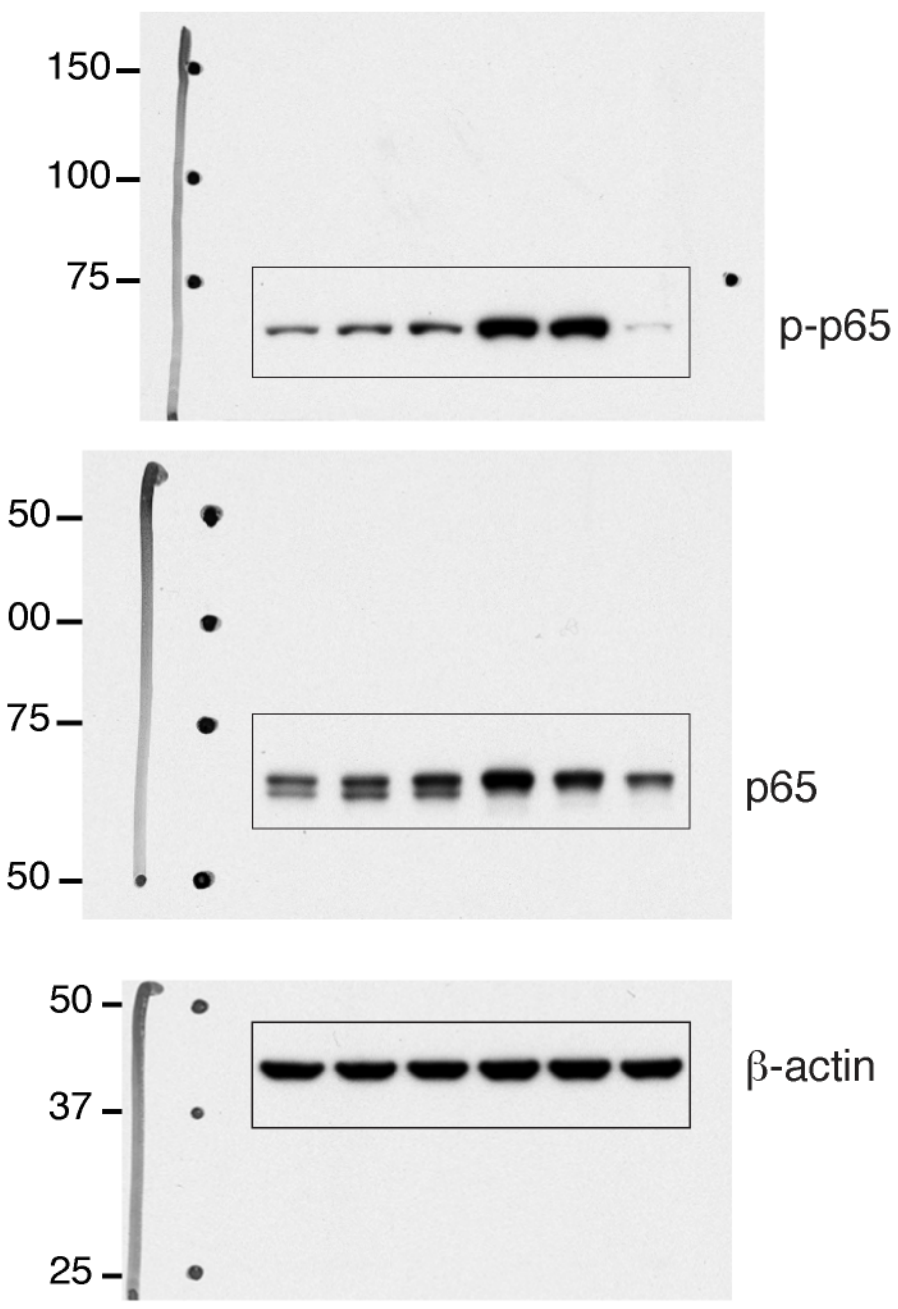

Figure 6b

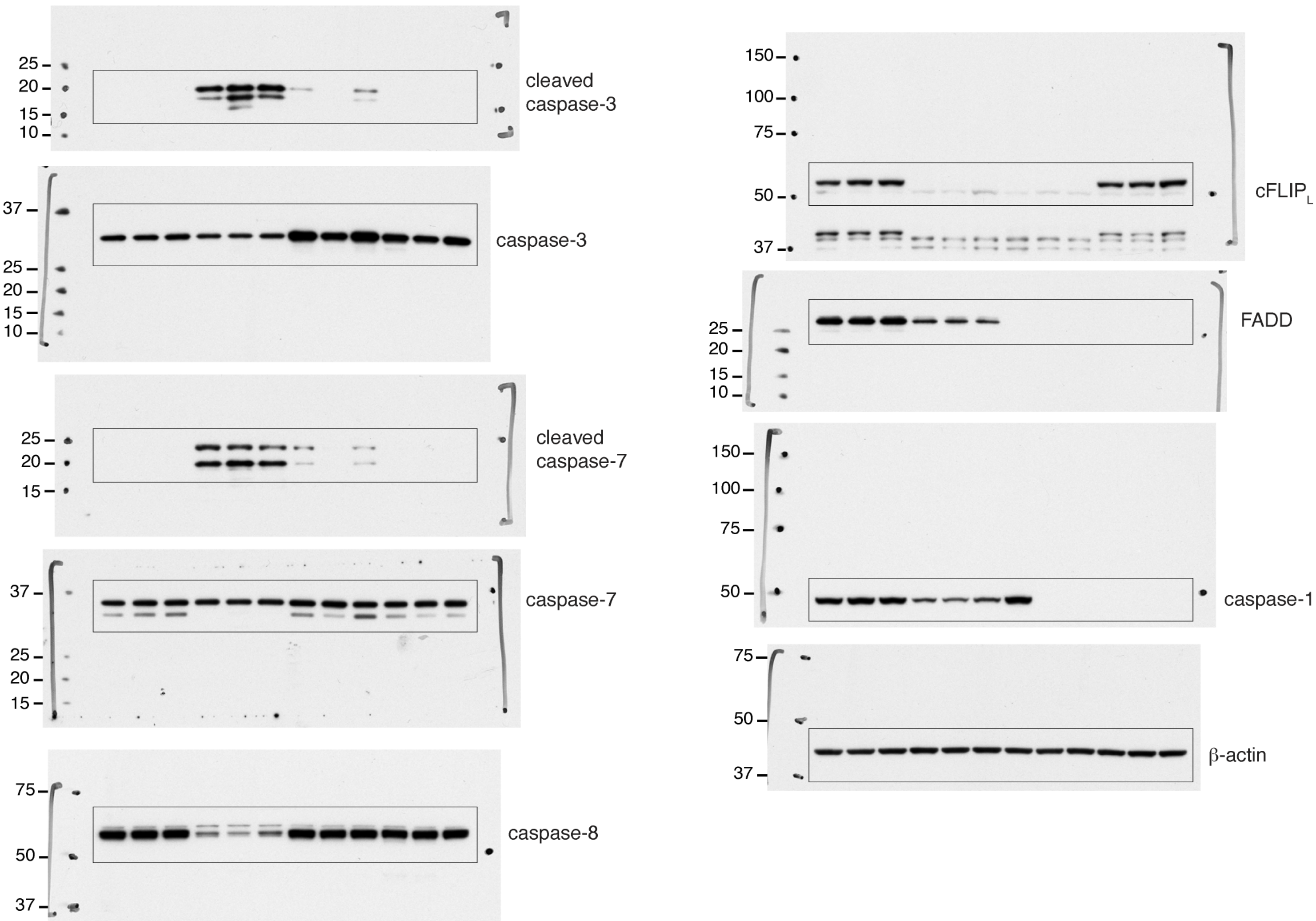

Figure S2

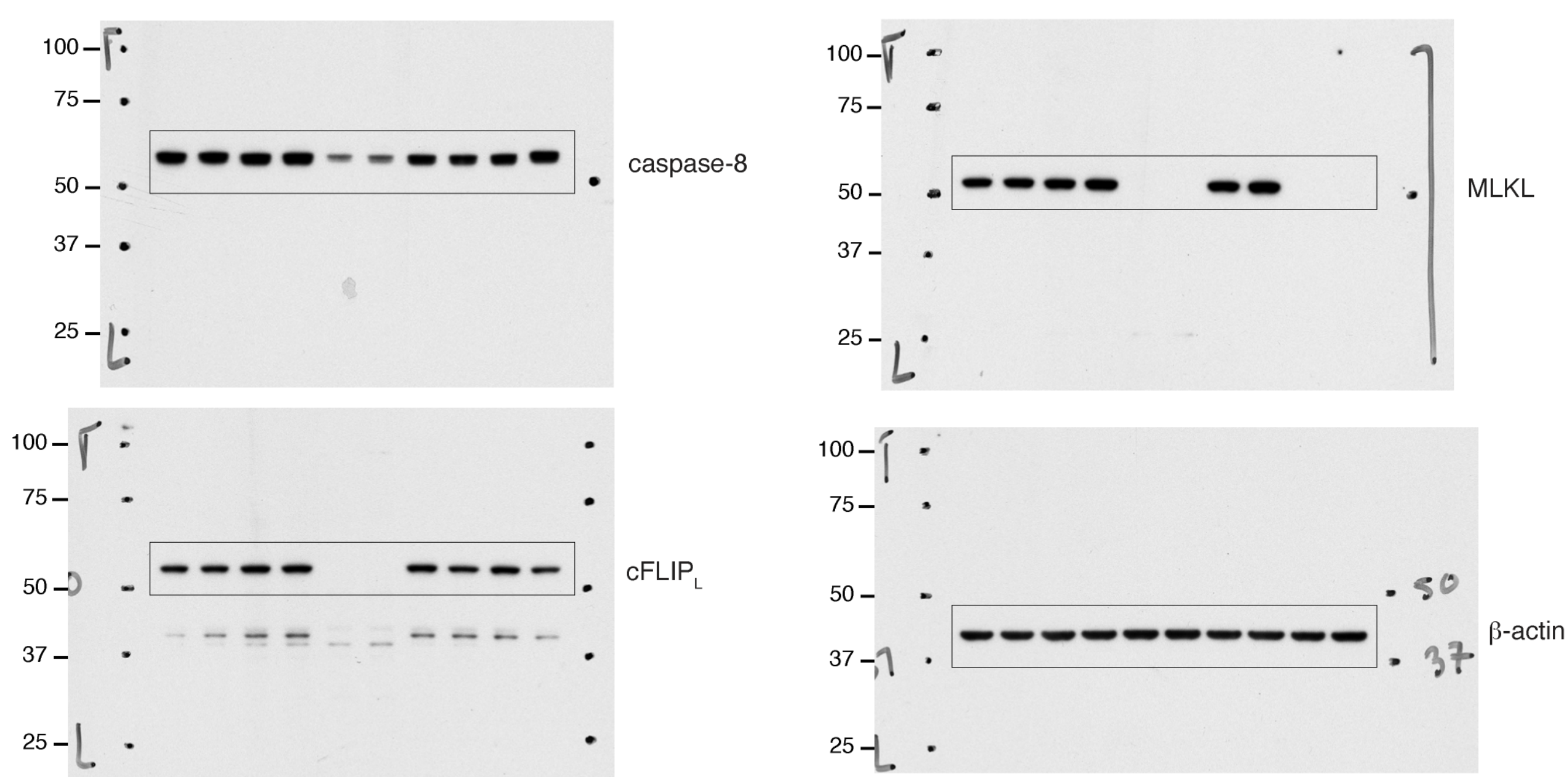

Figure S3a - WT and *Casp8*$^{1xDA/1xDA}$

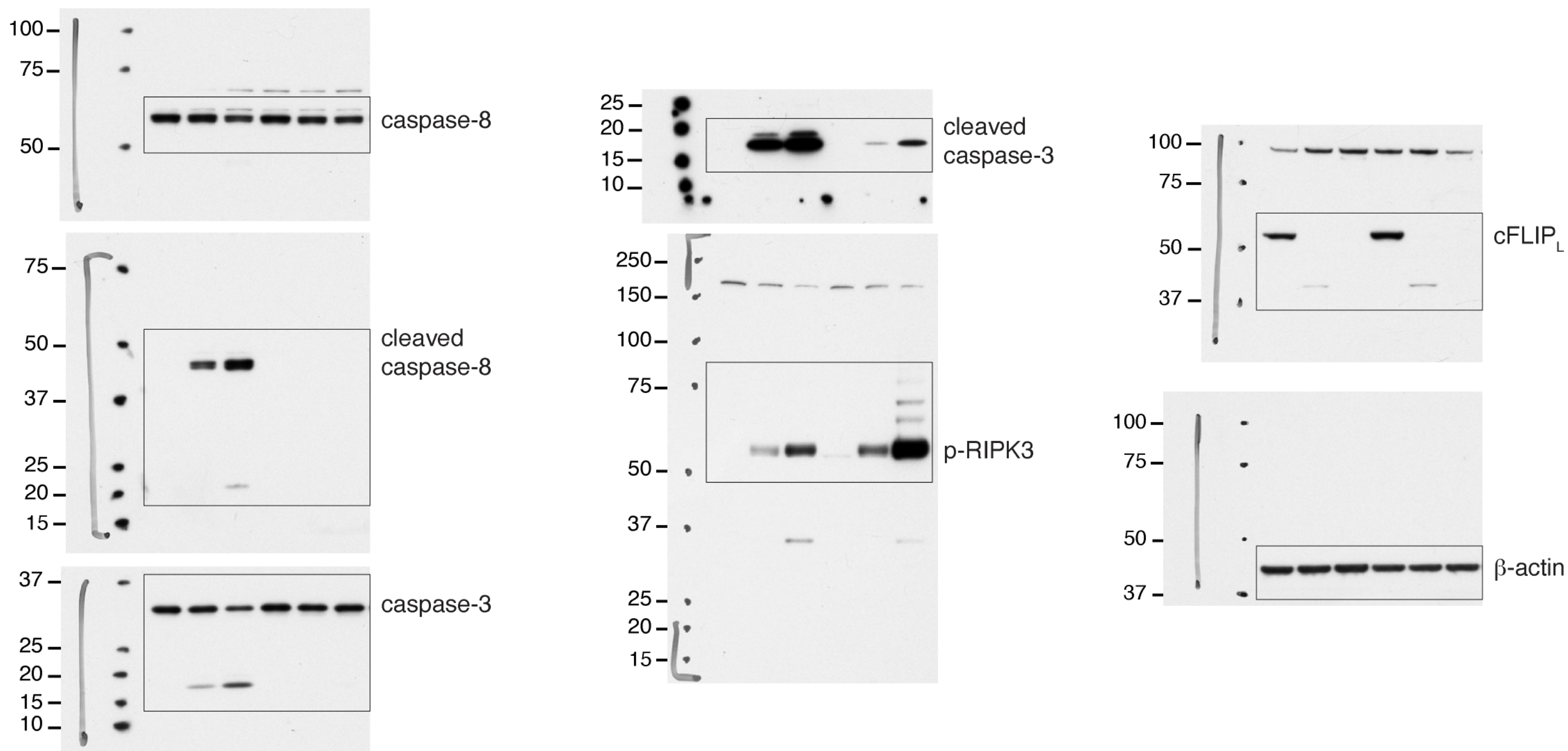

Figure S3a - WT and *Casp8*$^{4xDA/4xDA}$

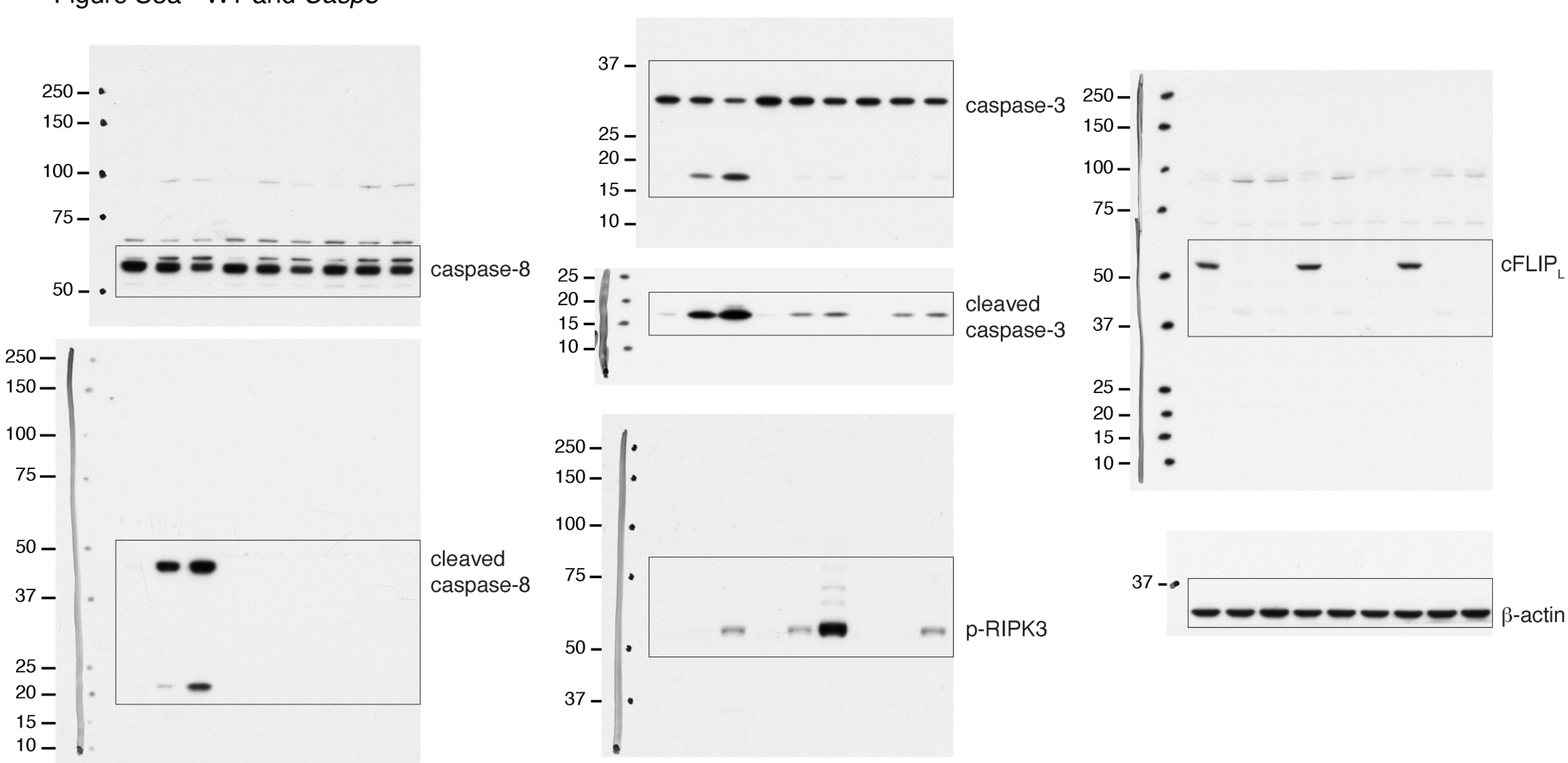

Figure S5a

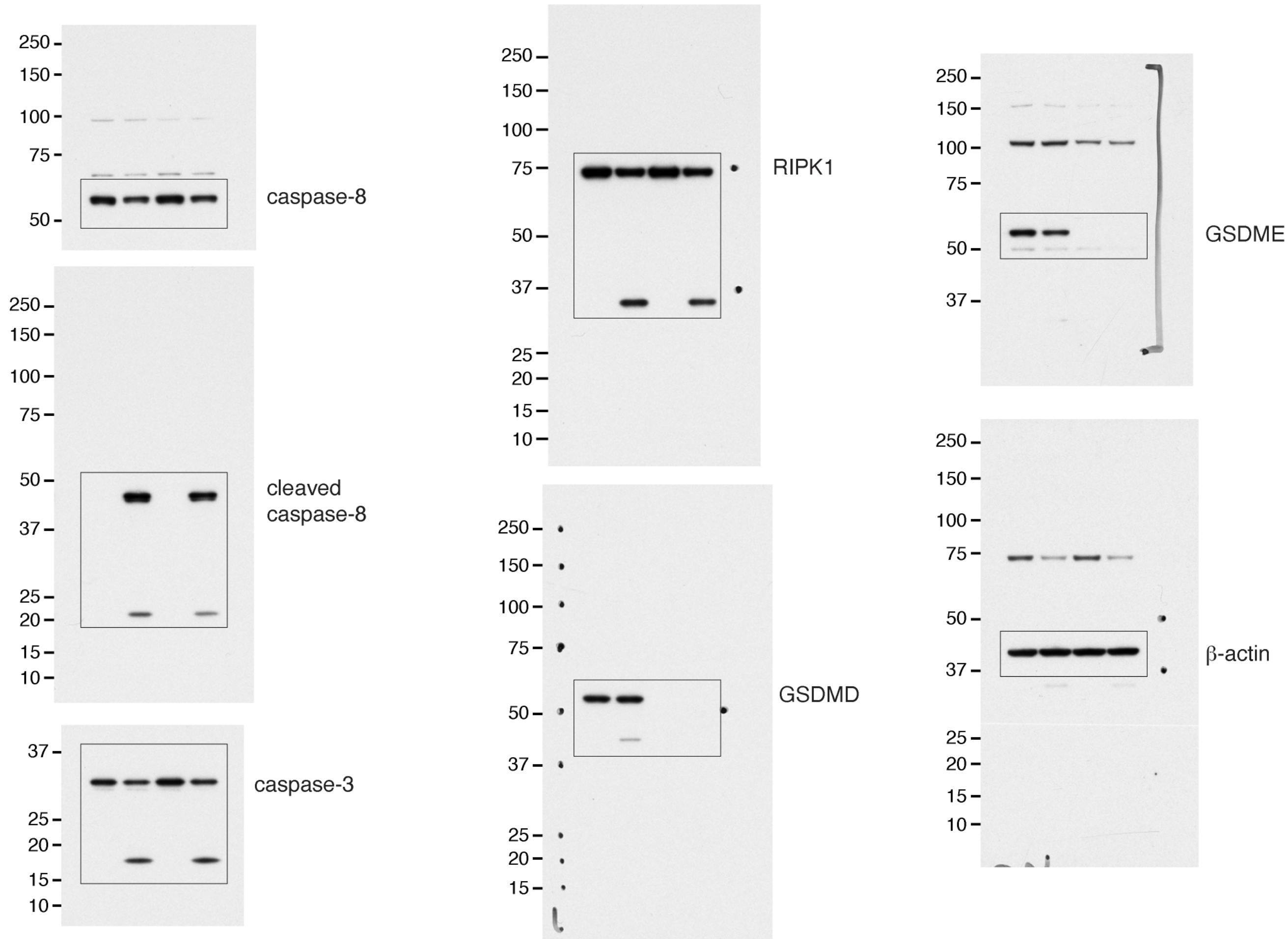

Figure S5d

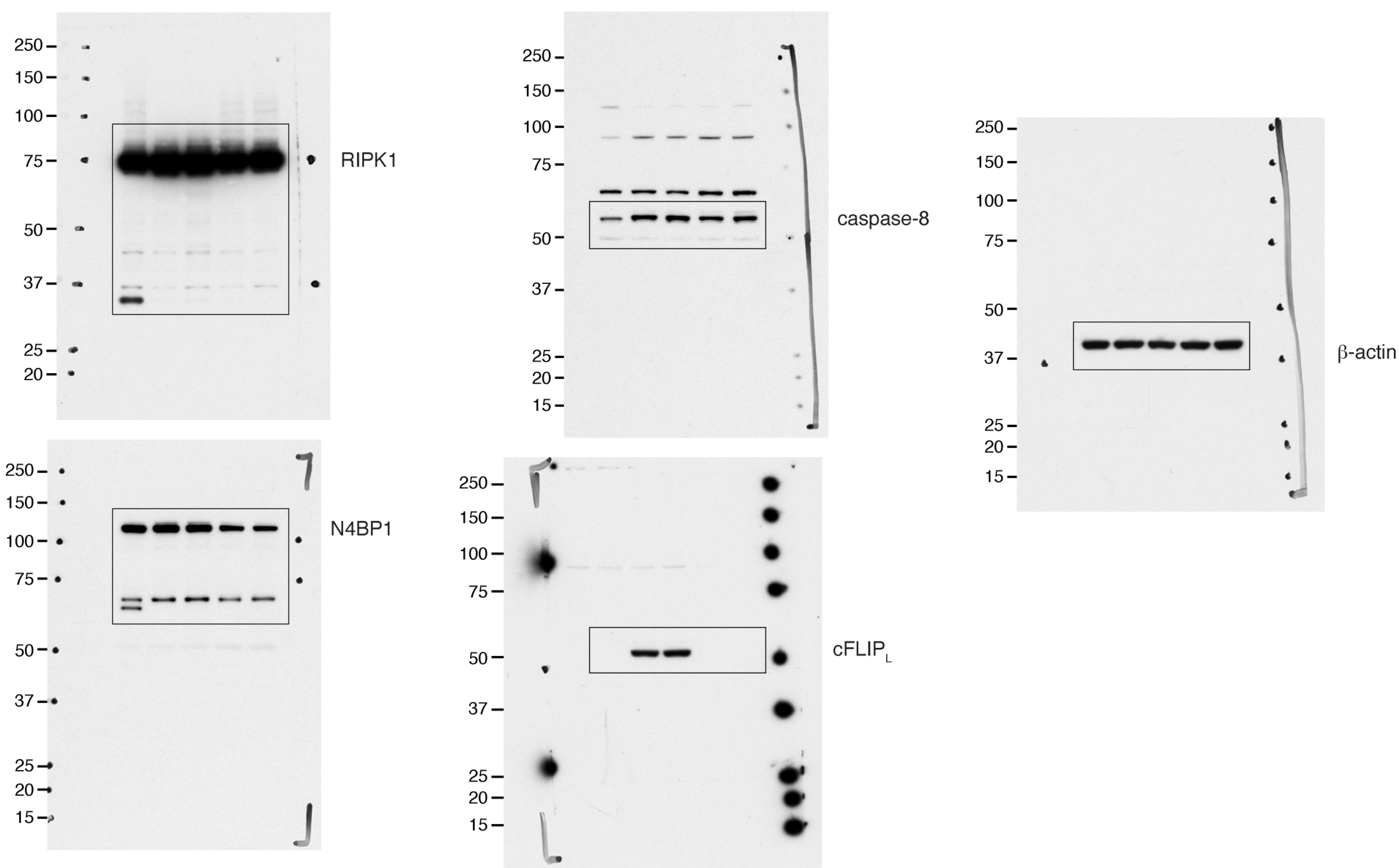

Figure S5e

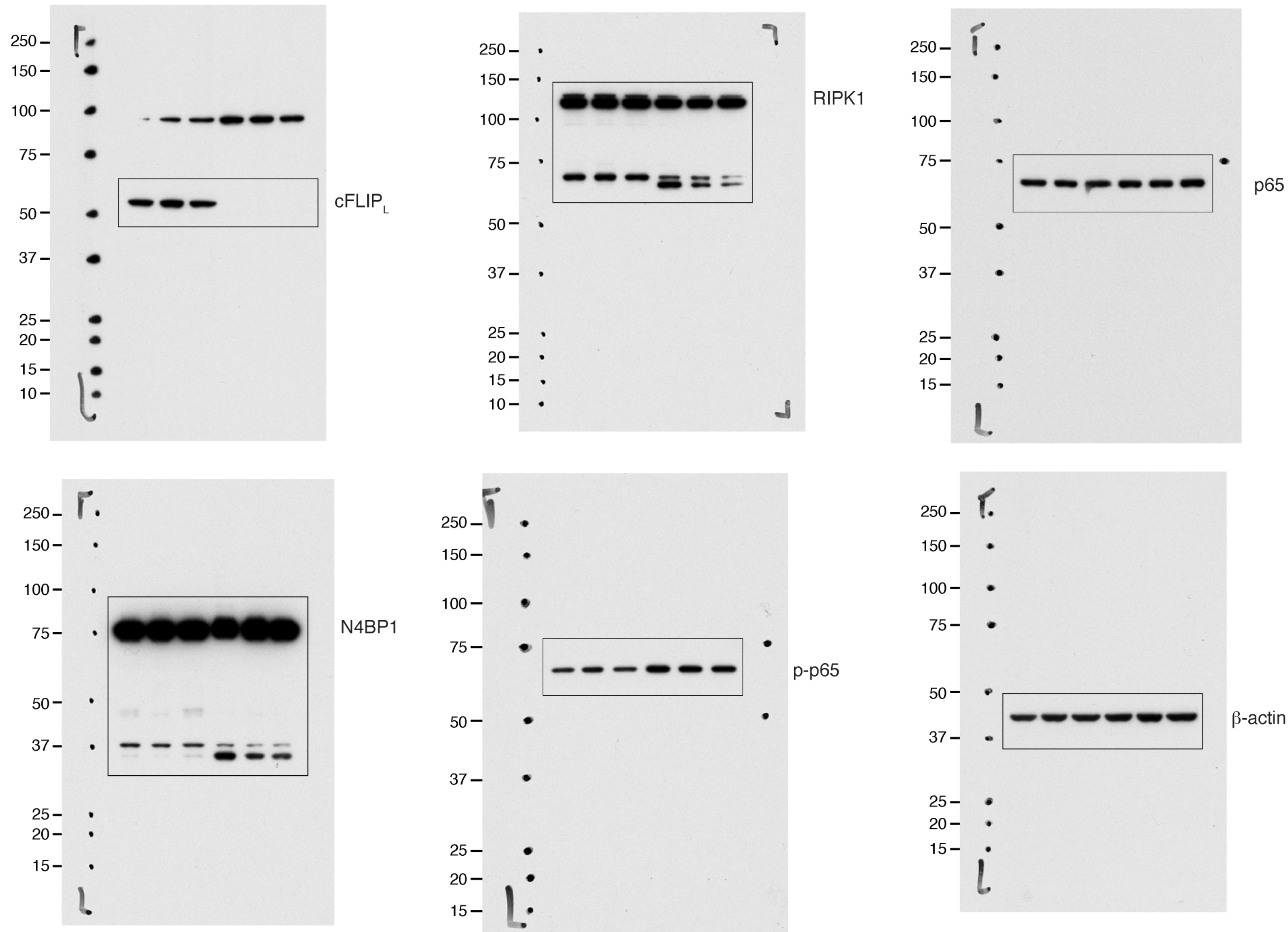
250
150
100
75
50
37
25
20
15
10
cFLIP$_L$
RIPK1
p65
N4BP1
p-p65
β-actin

Figure S5f

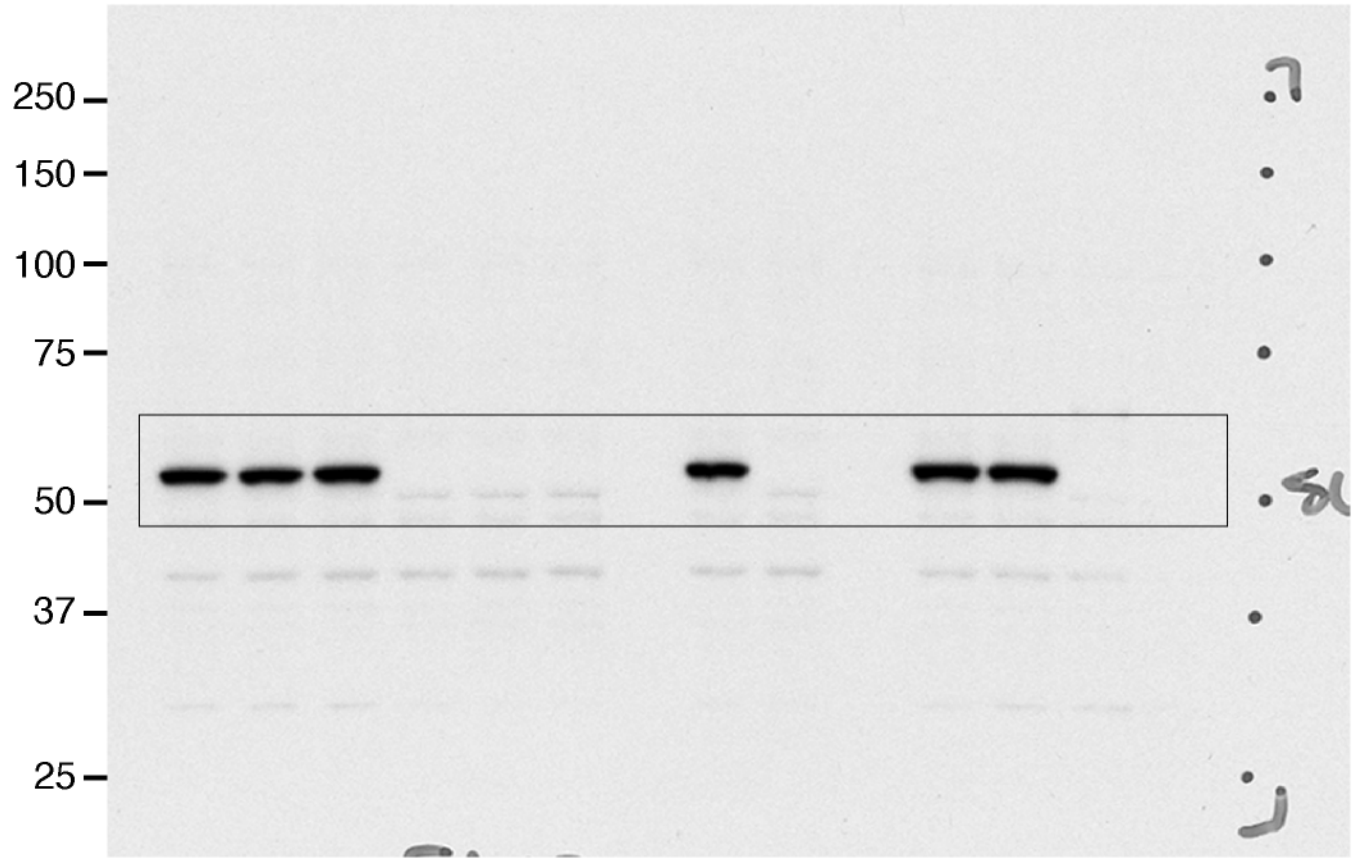

$cFLIP_L$

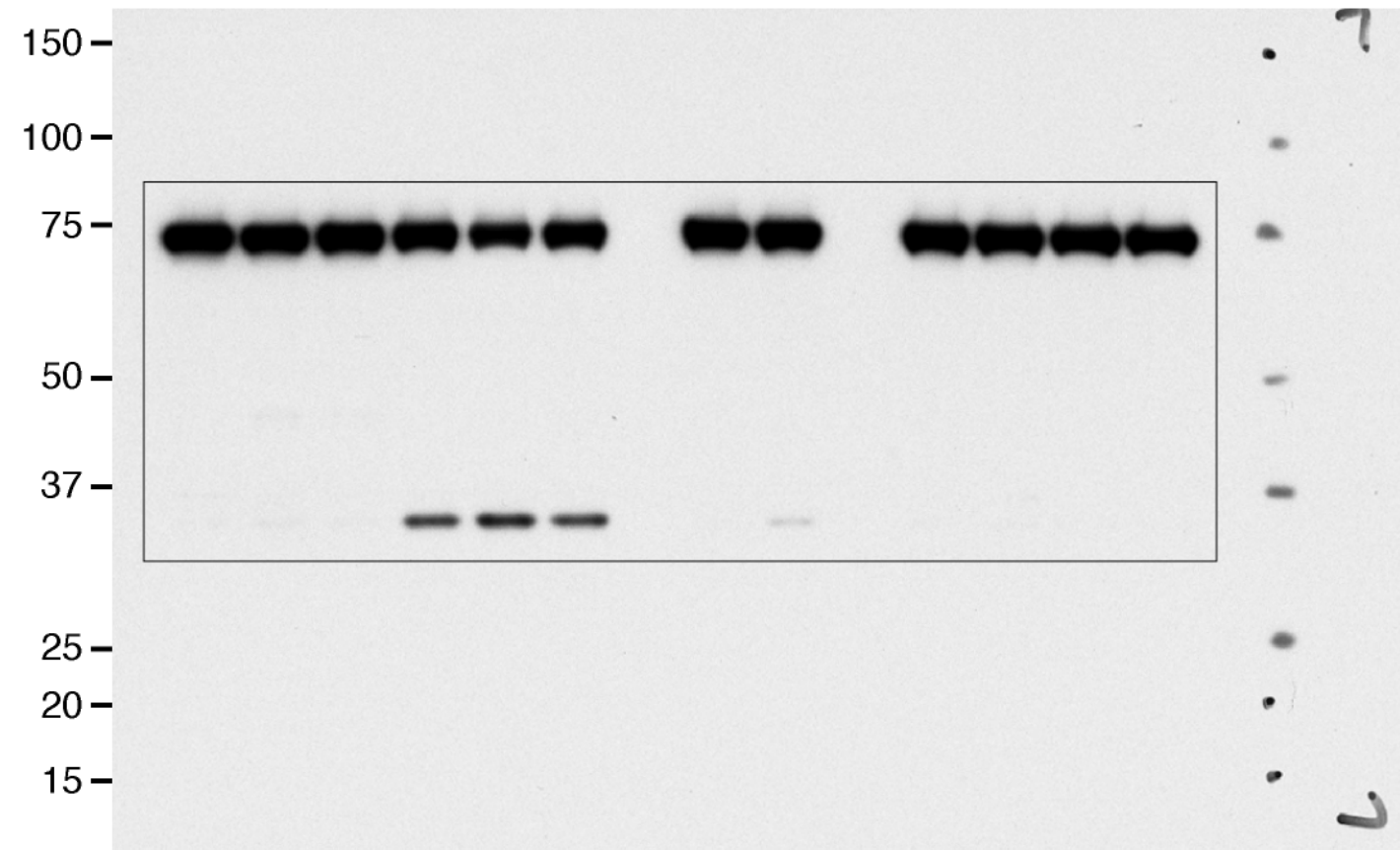

RIPK1

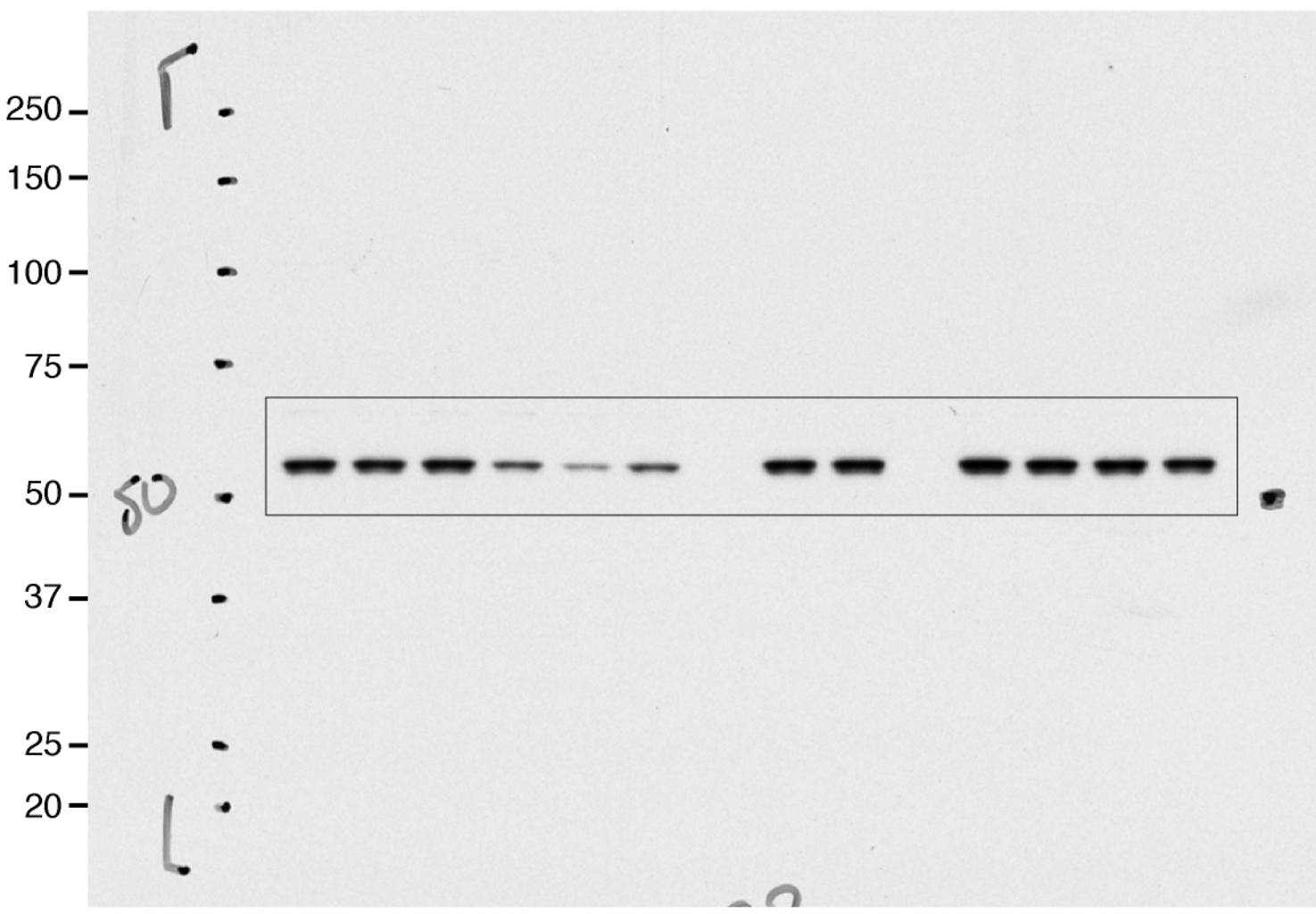

caspase-8

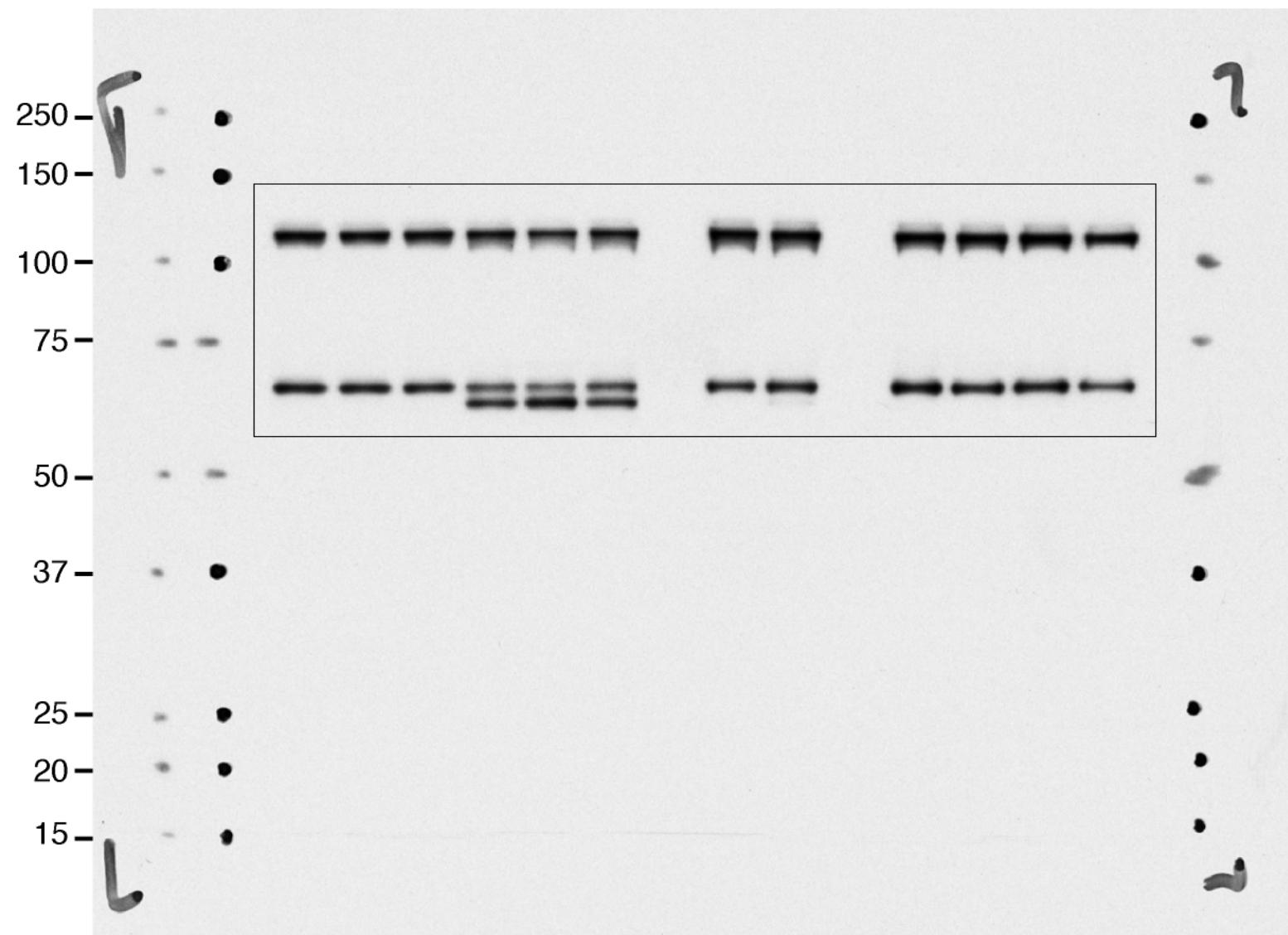

N4BP1

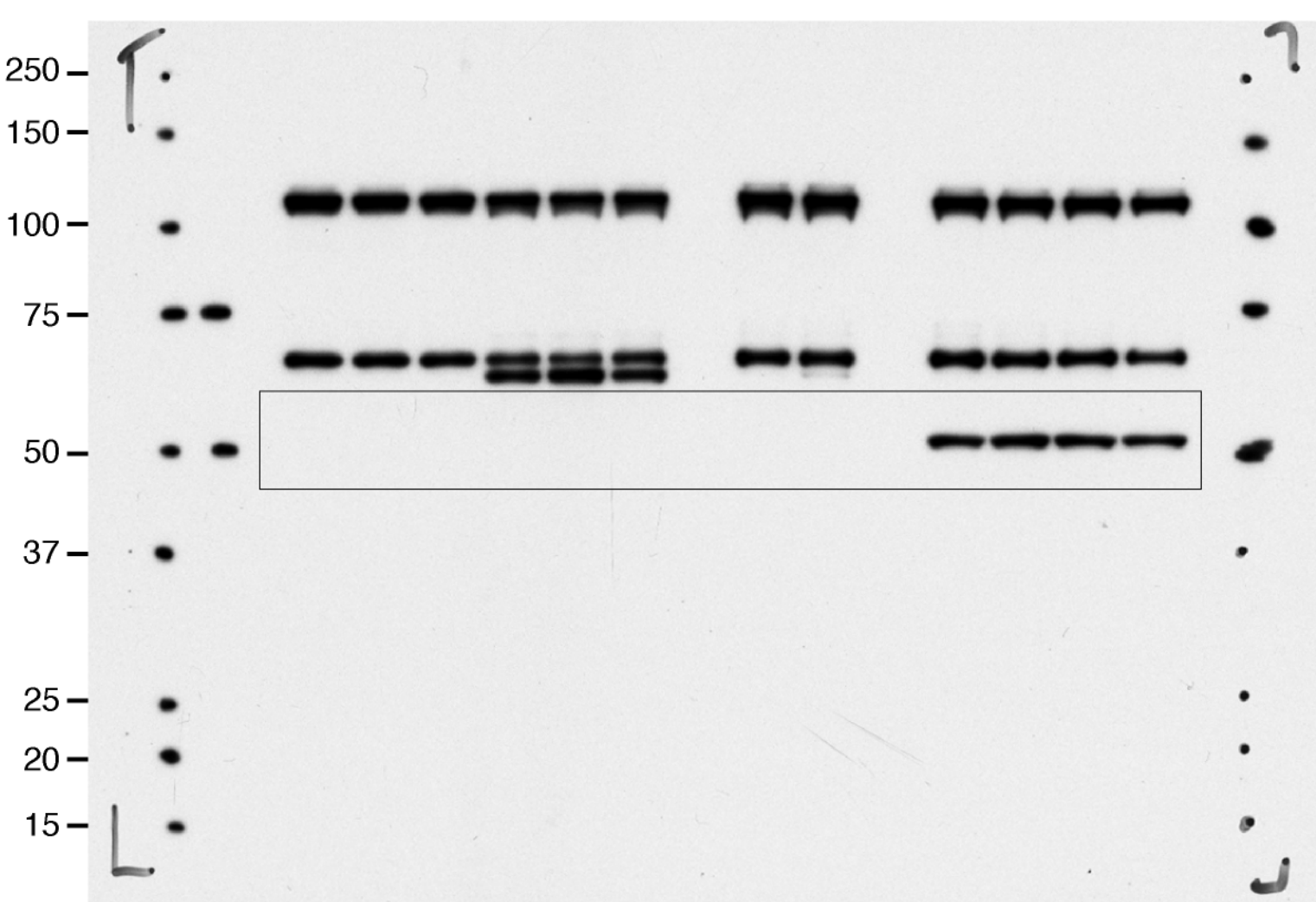

MLKL

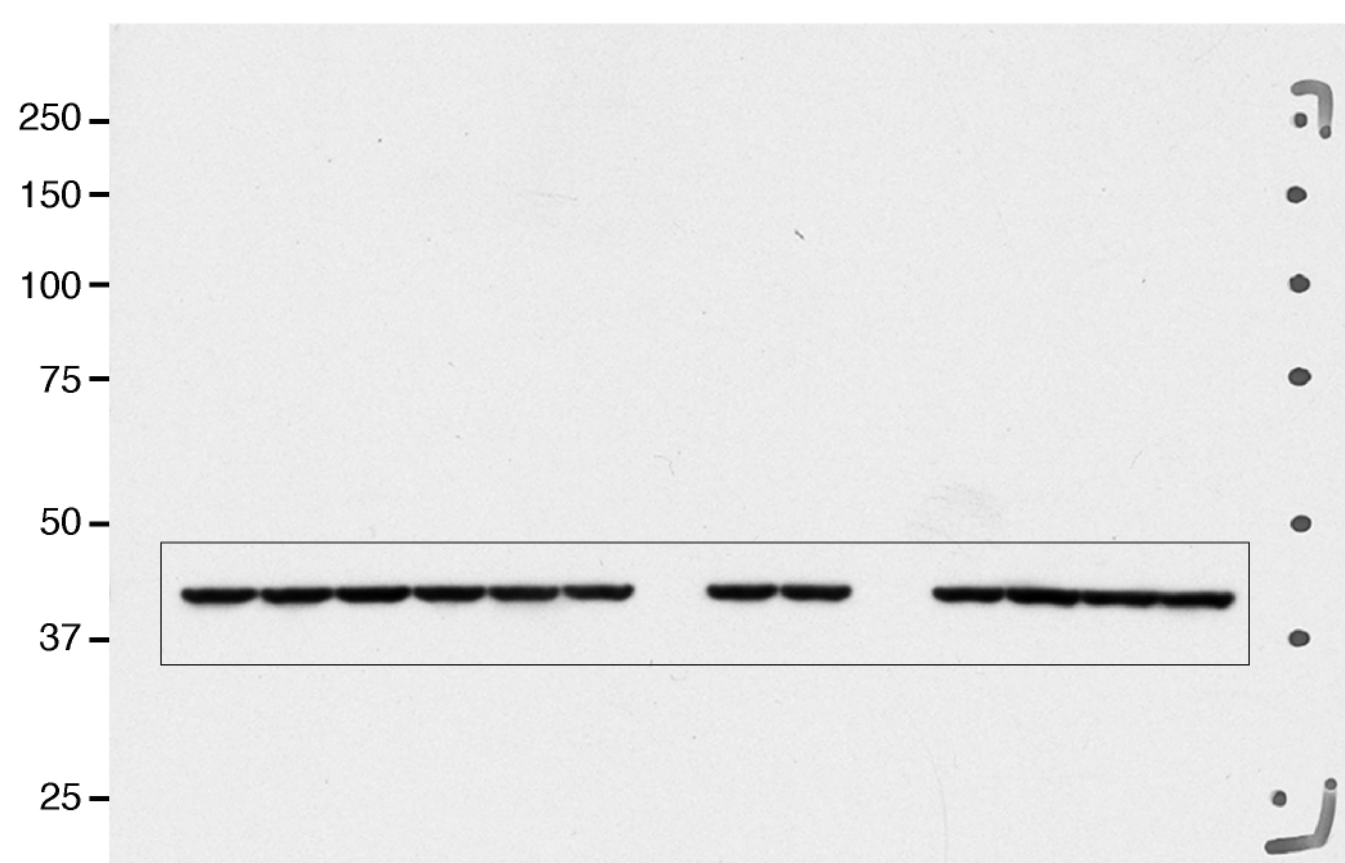

β-actin

Figure S5g

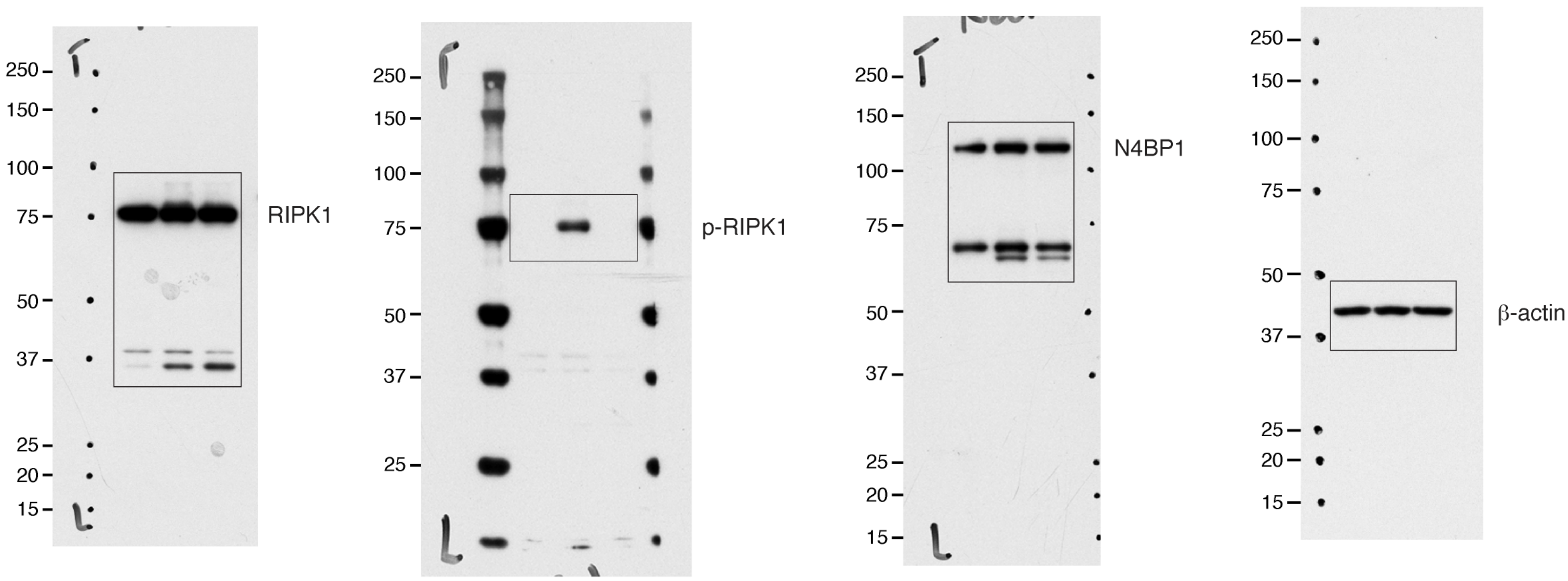

Figure S6a

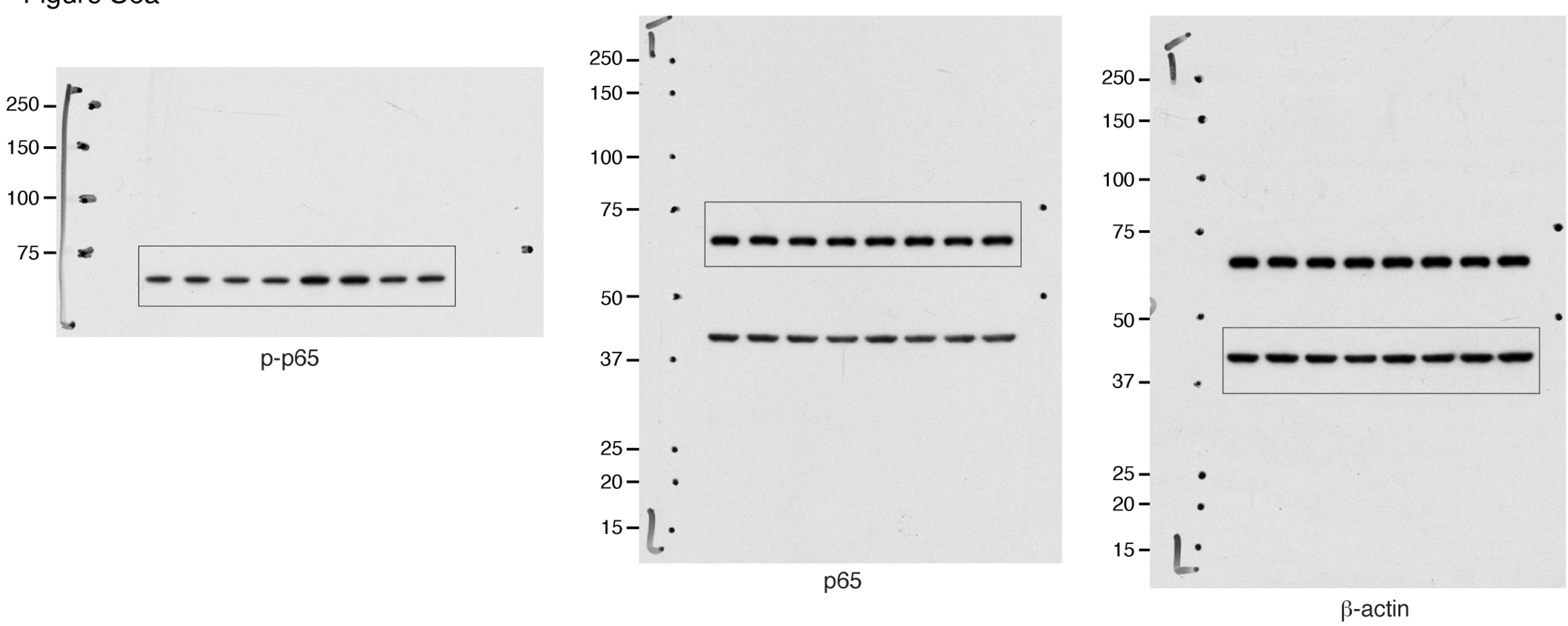

Supplement: Supplementary file 2 [file 41418_2025_1650_MOESM2_ESM.pdf]
